# Estimation of Directed Effective Connectivity from fMRI Functional Connectivity Hints at Asymmetries of Cortical Connectome

**DOI:** 10.1371/journal.pcbi.1004762

**Published:** 2016-03-16

**Authors:** Matthieu Gilson, Ruben Moreno-Bote, Adrián Ponce-Alvarez, Petra Ritter, Gustavo Deco

**Affiliations:** 1 Center for Brain Cognition, Dept of Technology and Information, Universitat Pompeu Fabra, Barcelona, Spain; 2 Research Unit, Parc Sanitari Sant Joan de Déu and Universitat de Barcelona, Esplugues de Llobregat, Barcelona, Spain; 3 Centro de Investigación Biomédica en Red de Salud Mental (CIBERSAM), Esplugues de Llobregat, Barcelona, Spain; 4 Department of Information and Communication Technologies, Universitat Pompeu Fabra, Barcelona, Spain; 5 Serra Húnter Fellow Programme, Universitat Pompeu Fabra, Barcelona, Spain; 6 Minerva Research Group Brain Modes, Max Planck Institute for Human Cognitive and Brain Sciences, Leipzig, Germany; 7 Department of Neurology, Charité - University Medicine, Berlin, Germany; 8 Bernstein Focus State Dependencies of Learning & Bernstein Center for Computational Neuroscience, Berlin, Germany; 9 Berlin School of Mind and Brain & Mind and Brain Institute, Humboldt University, Berlin, Germany; 10 Institució Catalana de la Recerca i Estudis Avançats, Universitat Barcelona, Barcelona, Spain; Oxford University, UNITED KINGDOM

## Abstract

The brain exhibits complex spatio-temporal patterns of activity. This phenomenon is governed by an interplay between the internal neural dynamics of cortical areas and their connectivity. Uncovering this complex relationship has raised much interest, both for theory and the interpretation of experimental data (e.g., fMRI recordings) using dynamical models. Here we focus on the so-called inverse problem: the inference of network parameters in a cortical model to reproduce empirically observed activity. Although it has received a lot of interest, recovering directed connectivity for large networks has been rather unsuccessful so far. The present study specifically addresses this point for a noise-diffusion network model. We develop a Lyapunov optimization that iteratively tunes the network connectivity in order to reproduce second-order moments of the node activity, or functional connectivity. We show theoretically and numerically that the use of covariances with both zero and non-zero time shifts is the key to infer directed connectivity. The first main theoretical finding is that an accurate estimation of the underlying network connectivity requires that the time shift for covariances is matched with the time constant of the dynamical system. In addition to the network connectivity, we also adjust the intrinsic noise received by each network node. The framework is applied to experimental fMRI data recorded for subjects at rest. Diffusion-weighted MRI data provide an estimate of anatomical connections, which is incorporated to constrain the cortical model. The empirical covariance structure is reproduced faithfully, especially its temporal component (i.e., time-shifted covariances) in addition to the spatial component that is usually the focus of studies. We find that the cortical interactions, referred to as effective connectivity, in the tuned model are not reciprocal. In particular, hubs are either receptors or feeders: they do not exhibit both strong incoming and outgoing connections. Our results sets a quantitative ground to explore the propagation of activity in the cortex.

## Author Summary

The study of interactions between different cortical regions at rest or during a task has considerably developed in the past decades thanks to progress in non-invasive imaging techniques, such as fMRI, EEG and MEG. These techniques have revealed that distant cortical areas exhibit specific correlated activity during the resting state, also called functional connectivity (FC). Moreover, recent studies have highlighted the possible role of white-matter projections between cortical regions in shaping these activity patterns. This structural connectivity (SC) can be estimated using MRI, which measures the probability for two areas to be connected via the density of neural fibers. However, this does not provide the strengths of dynamical interactions. Many methods have thus been developed to estimate the connectivity between neural populations in the cortex that is hypothesized to shape FC. The strengths of these dynamical interactions are called effective connectivity (EC). We use a cortical model that combines information from Diffusion-weighted MRI (dwMRI) and fMRI in order to estimate EC. We demonstrate theoretically that directed *C* can be inferred using time-shifted covariances. The key point of our method is the use of temporal information from FC at the scale of the whole network. Applying our model on experimental fMRI data at rest, we estimate the asymmetry of intracortical connectivity. Obtaining an accurate EC estimate is essential to analyze its graph properties, such as hubs. In particular, directed connectivity links to the asymmetry between input and output EC strengths of each node, which characterizes feeder and receiver hubs in the cortical network.

## Introduction

Patterns of neural activity at the scale of the whole cortex can be quantified by the correlations between the cortical regions. This so-called functional connectivity (FC) is predicted to some extent by the anatomical synaptic pathways in the white matter, or structural connectivity (SC) [1]. However, SC measures the neural fiber density and is not sufficient to fully explain the structure of FC, which also depends on the dynamics of network nodes. The dynamical interactions between cortical areas is captured by the ‘effective connectivity’ (EC), a concept that has emerged over years following progress in imaging techniques and related modeling [2–5]. In network models, EC is the key to understand the propagation of information, which links the network structure to function. Importantly, EC is model-dependent, which makes the comparison between studies non trivial and has raised much debate recently [6–8]. On the biological ground, EC accounts for mechanisms that determine the synaptic strength (e.g., types and concentration of neurotransmitters), as well as dynamic properties such as neural excitability that may vary with the local activity level and thus depend on the inputs to the network. This means that EC may differ from the anatomical SC obtained from dwMRI. FC and EC classically relate to data and models related to imaging techniques: fMRI, EEG and MEG. Beyond the analysis of these experimental data, uncovering the relationship between connectivity and activity has attracted much interest recently, with a particular focus for non-trivial connectivity topologies that mimic those observed in the biology [9–12]. Within this field, the inference of the network connectivity from empirically observed activity is an active line of research [1, 13–16] and is the purpose of the present study.

In order to infer the connectivity from empirical observations, one needs to define observables of the activity that the network model has to reproduce. Designing adequate and efficient estimation procedures is as important as the properties of the model itself. Over the past years, many techniques have been developed for various dynamical models: for oscillator networks based on synchrony [17] and covariances [18, 19]; structural equation models (SEMs) [20, 21]; statistical methods for generalized linear models (GLMs) [13, 22]; Granger causality in spiking networks with nonlinear dynamics [14, 23]; transfer entropy for an abstract model of neural activity observed via calcium imaging [24]; spike-time covariances in networks of Poisson neurons [25]; covariances for multivariate Ornstein-Uhlenbeck processes [16] with recent developments to estimate directed connectivity [26]; for the dynamic causal model (DCM) [5, 15]. As a general rule, three factors limit the accuracy of connectivity estimation: the size of the network, the amount of empirically observed activity and nonlinearities in the mapping between the connectivity and the activity observables. Moreover, the directionality of interactions between nodes is harder to estimate than the existence of an interaction between two nodes.

Here we do not focus on the detection of connections, which often involves statistical tests, but we want to estimate the ranking of individual weights in networks of about a hundred of nodes. In Methods, we develop the theory to recover from observed covariances (taken as FC) the network connectivity as well as the intrinsic variability experienced by individual nodes in a noise-diffusion (ND) network. More precisely, we perform a Lyapunov optimization (LO) during which the network parameters are tuned iteratively such that the network reproduces two covariance matrices set as ‘objectives’, or goals. This allows for the use of constraints on the network parameters, such as enforcing partial connectivity. The model is a multivariate Ornstein-Uhlenbeck process, where the activity fluctuations are caused by the intrinsic noise and shaped by the recurrent connections to generate the spatio-temporal covariance pattern. Compared to previous studies [18], we focus on the situation where the network effect is strong, meaning that FC significantly differs from EC: even unconnected or weakly connected nodes can be strongly correlated. This situation was considered for symmetric EC [27, 28]. As reviewed in [15] for the DCM model, many methods only consider connections individually for the observables; in contrast, partial correlations and multivariate autoregressive models (MVAR) consider the entire network activity and give the best performance for detecting undirected connections in that study. Our method also relies on the network covariances as a whole to estimate the connectivity. The performance of the estimation procedure for artificial networks is the subject of the first half of Results.

Following previous studies [1, 3, 5, 27, 29], we apply our theory to the estimation of cortico-cortical connectivity from fMRI data. Obtaining quantitative estimates for the connectivity is important to gain insight on the information flow within the cortex [30]. In our ND model, EC coincides with the recurrent connectivity. In many models applied to fMRI data such as the DCM [5] and dynamic mean-field (DMF) model [1], the hemodynamic response function (HRF) transforms the neural activity into the BOLD signal, introducing nonlinearity in the mapping EC-FC. Our ND model does not explicitly incorporate hemodynamics, so the estimated EC describes the interactions between cortical regions via the proxy of the BOLD signals. Therefore, we test our method on the more elaborate DMF model that incorporates a HRF [27]. Specifically, we verify that the spatio-temporal covariances in the DMF model with time shifts of seconds convey information about the underlying neural connectivity, which is required for our method to be successfully applied to fMRI data. Then, we check the agreement of the EC estimated using the ND model coincides with the neural connectivity of the DMF model. Finally we examine the consistency of our results when varying optimization parameters, in particular the time shift for the objective covariance matrices. In several previous models of the whole cortex [12, 27, 28, 31], EC corresponds to a scaled version of SC matrix obtained from diffusion tensor imaging, which is quasi symmetric. The corresponding fitting procedures often rely on zero-time-shift correlations, which implies that the directionality of cortical connections can hardly be estimated. In contrast, another direction of research has investigated directed interactions, but only for a small number of cortical areas [2–5, 7, 20, 32]. By recovering the strengths of individual connections and intrinsic variability for the whole cortex divided in 68 areas, we aim to combine the advantages of those studies.

## Methods

We present the theory used to go back and forth between the network parameters and the observables:

- the forward problem: calculation of theoretical covariance matrices *Q*^0^ and *Q*^*τ*^ for given matrices for the network connectivity *C* and intrinsic noise Σ;
- the inverse problem: estimation of *C* with fixed Σ and estimation of both *C* and Σ when the *Q* matrix(ces) is(are) known.

The variables of importance are described in Table 1.

**Table 1:** Table of variables used in Methods and Results.

Variable name	Symbol
node activity	$x_{i}^{t}$
model network connectivity	*C*
noise matrix (intrinsic variability)	Σ
model zero-time-shift covariances	*Q*^0^
model time-shifted covariances	*Q*^*τ*^
objective zero-time-shift covariances	${\hat{Q}}^{0}$
objective time-shifted covariances	${\hat{Q}}^{\tau }$

**Fig 1:**
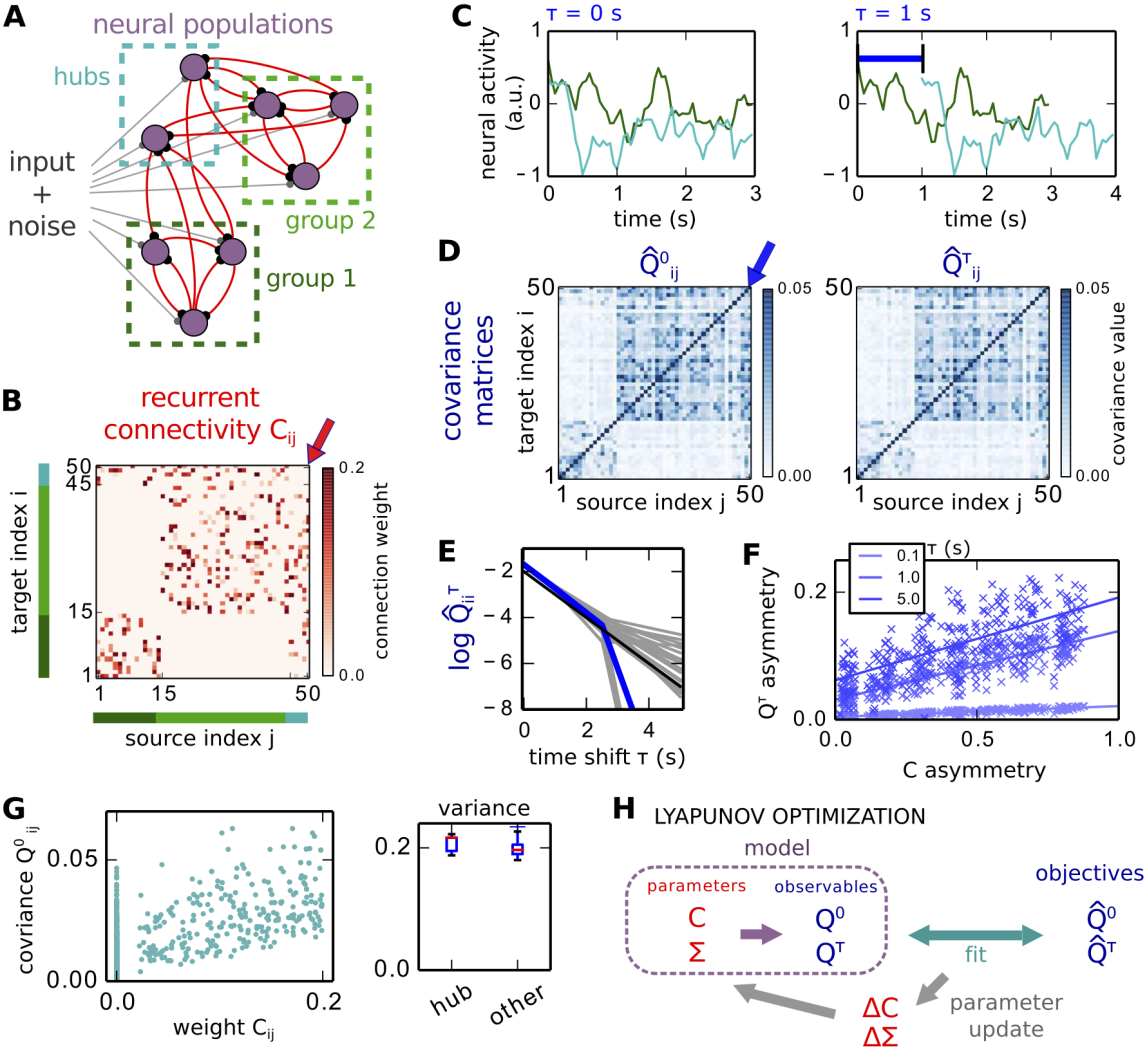
Network of interacting neural populations. **A** Schematic diagram of the ND model: the neural populations (purple circles) receive noisy inputs as well as recurrent feedback (gray and red connections, respectively). The topology is non-random: 2 groups of interconnected populations (green dashed boxes) are linked by hubs (cyan dashed box), but the hubs are not connected to each other. **B** Connectivity matrix *C* for a network of 50 populations, corresponding to the strengths of the red connections in A. The indices for the two groups are indicated by the green bars, and hubs by the cyan bar. **C** Example time series between two populations in the network, one from the large group in A and a hub. In the right panel, the darker curve is shifted by *τ* = 1 s. **D** Empirical covariance matrices between all neural populations, evaluated from the time series in C with the two time shifts: *τ* = 0 for ${\hat{Q}}^{0}$ and *τ* = 1 s for ${\hat{Q}}^{\tau }$; see expression for *Q*^*τ*^ in Eq (2). The color scale is adjusted to focus on off-diagonal elements. **E** Logarithm of autocovariance ${\hat{Q}}_{ii}^{\tau }$ for each node *i* as a function of *τ*. The mean over all nodes (blue curve) has a slope close to 1/*τ*_*x*_ (black line). **F** Asymmetry of theoretical *Q*^*τ*^ as a function of the asymmetry of *C*. The asymmetry index in Eq (24) scales from 0 for symmetric to 1 for antisymmetric matrices. Three time shifts *τ* are displayed (lighter to darker blue), each cross represents a cluster-hub network similar to A and the corresponding line indicates the linear fit. **G** Network effect indicated by the weak match between *C* weights and *Q*^0^ values over all connections. **H** Schematic representation of a step in the Lyapunov optimization (LO) procedure. The model covariances *Q*^0,*τ*^ are evaluated for the current network parameters *C* and Σ. From the comparison between the model *Q*^*τ*/0^ and their objective counterparts ${\hat{Q}}^{\tau /0}$, the desired updates Δ*C* and ΔΣ for the model parameters are calculated.

We consider a network of interconnected neural populations, as schematized in Fig 1A. The matrix *C* in Fig 1B represents the connection strengths for such a network of size 50. In our model, each node receives noise that propagates due to the recurrent connectivity. The activity in population *i* is described by the variable $x_{i}^{t}$, where *t* denotes the time. An example for the activity of two nodes is displayed in the left graph of Fig 1C. We analyze the neural dynamics up to the second-order fluctuations, assuming stationarity of the whole stochastic process. The means ${\bar{x}}_{i}$ and covariances $Q_{ij}^{\tau }$ of variables $x_{i}^{t}$ are defined as:

$$\begin{array}{ll} \bar{x} & :=\langle x_{i}^{t}\rangle ,\\ Q_{ij}^{\tau } & :=\langle \left(x_{i}^{t}-{\bar{x}}_{i}\right)(x_{j}^{t+\tau }-{\bar{x}}_{j})\rangle , \end{array}$$ (1)

where the angular brackets correspond to the average over the randomness due to the noise. The empirical covariances are evaluated from the discrete time series $x_{i}^{n,t}$ with the corresponding time shifts from the activity of *n*_*sim*_ simulated sessions with *T* samples each:

$${\hat{Q}}_{ij}^{\tau }=\frac{1}{n_{\text{sim}}T}\sum_{\begin{array}{c} 1\leq n\leq n_{\text{sim}}\\ 0\leq t<T \end{array}}\left(x_{i}^{n,t}-{\hat{x}}_{i}^{n}\right)\left(x_{j}^{n,t+\tau }-{\hat{x}}_{j}^{n}\right),$$ (2)

where the means ${\hat{x}}_{i}^{n}=\sum_{0\leq t<T}x_{i}^{n,t}/T$ are evaluated for each session. The stationarity hypothesis implies that averaging ${\hat{Q}}_{ij}^{\tau }$ over for a sufficiently long period gives the probabilistic mean $Q_{ij}^{\tau }$. For the time series in Fig 1C, we obtain the ${\hat{Q}}^{0}$ and ${\hat{Q}}^{\tau }$ matrices in Fig 1D. By definition, the matrix *Q*^*τ*^ is symmetric for *τ* = 0, but its asymmetry increases with *τ*, as shown by lighter plotted dots and fitting curves in Fig 1F.

### Model of interconnected cortical areas

The node activity is governed by the following coupled ordinary differential stochastic equations:

$$dx_{i}^{t}=\left[-\frac{x_{i}^{t}}{\tau_{x}}+\sum_{k\neq i}C_{ik}x_{k}^{t}+e_{i}\right]dt+dB_{i}^{t}\;.$$ (3)

Each $x_{i}^{t}$ experiences an exponential decay with time constant *τ*_*x*_ and is excited by other $x_{k}^{t}$ whose contributions are scaled by the recurrent weights *C*_*ij*_. Fluctuations are generated by the Gaussian white noise $dB_{i}^{t}$ that has variance (*σ*_*i*_)^2^; formally, $B_{i}^{t}$ is a Wiener process scaled by a factor *σ*_*i*_. We forbid self-connections, i.e., *C*_*ii*_ = 0, although this is not crucial. In practice and without loss of generality, the input *e*_*i*_ is homogeneous for all nodes.

Following previous work [16, 27, 33], we derive the well-known self-consistency equations for the mean $\bar{x}$ and covariances *Q*^*τ*^ in Eq (1), assuming stationarity of the process. When the system has a stable fixed point, it is given by the zeros of the linear matrix system:

$$J\bar{x}+e=0\;,$$ (4)

where *x*^*t*^ indicates the activity vector whose entries are $x_{i}^{t}$ for 1 ≤ *i* ≤ *N*; *e* is the homogeneous vector of inputs *e*_*i*_. We have denoted the Jacobian of Eq (3) by

$$J_{ij}=\frac{-\delta_{ij}}{\tau_{x}}+C_{ij}\;,$$ (5)

where the Kronecker delta *δ*_*ij*_ = 1 if *i* = *j* and 0 otherwise. The system has a single fixed point provided *J* is invertible.

Using Ito’s formula for the derivation of $\left(x_{i}^{t}-{\bar{x}}_{i})(x_{j}^{t+\tau }-{\bar{x}}_{j}\right)$ with respect to *t*, we obtain the Lyapunov equation for the steady-state of the second-order fluctuations with zero time shift:

$$JQ^{0}+Q^{0}J^{†}+\Sigma =0.$$ (6)

The noise matrix Σ is diagonal with terms ${\left(\sigma_{i}\right)}^{2}=\delta_{ij}\langle dB_{i}^{t}dB_{j}^{t}\rangle$ and † is the matrix transpose operator.

As done for the variable *t*, the derivation of $\left(x_{i}^{t}-{\bar{x}}_{i})(x_{j}^{t+\tau }-{\bar{x}}_{j}\right)$ with respect to *τ* yields an equation satisfied by the time-shifted covariance *Q*^*τ*^:

$$Q^{\tau }=Q^{0}\;\text{expm}\left[J^{†}\tau \right]\;,$$ (7)

where **expm** is the matrix exponential. When *J* is dominated by the diagonal elements, the autocovariances $Q_{ii}^{\tau }$ are close to an exponential decay with time constant *τ*_*x*_, as illustrated in Fig 1E with a log y-axis by the straight black line that coincices with the mean in blue up to *τ* = 2.5 s.

### Evaluating covariances from known network parameters

For a model with given connectivity matrix *C* and intrinsic noise matrix Σ, the theoretical zero-time-shift covariance matrix *Q*^0^ is the unique solution of Eq (6) and can be evaluated using the long-known Bartels-Stewart algorithm [16]. Then, the model *Q*^*τ*^ can be calculated from *Q*^0^ and *J* using Eq (7) for any given *τ*.

### Direct estimation of network parameters from empirical covariances

The inverse problem consists in finding an estimate of the connectivity matrix *C* such that the model reproduces the two objective matrices ${\hat{Q}}^{0}$ and ${\hat{Q}}^{\tau }$ for a given *τ* > 0, e.g., given by empirical observations. The relation in Eq (7) allows for the calculation of *J* from ${\hat{Q}}^{0}$ and an occurrence of ${\hat{Q}}^{\tau }$ for a given *τ* > 0:

$$J=\frac{1}{\tau }\;{\left\{\text{logm}\left[{\left({\hat{Q}}^{0}\right)}^{-1}{\hat{Q}}^{\tau }\right]\right\}}^{†}\;,$$ (8)

where **logm** is the matrix logarithm [34]. In our case, the non-diagonal elements of the Jacobian directly give the connectivity weight *C*_*ij*_ = *J*_*ij*_. Note that the diagonal terms of *J* can provide an estimation of *τ*_*x*_, so far as we assume *C*_*ii*_ = 0. Then, the noise matrix can be evaluated from the Lyapunov equation Eq (6):

$$\Sigma =-J{\hat{Q}}^{0}-{\hat{Q}}^{0}J^{†}.$$ (9)

Due to the matrix logarithm, this method is very sensitive to the noise in ${\hat{Q}}^{0}$ and ${\hat{Q}}^{\tau }$: it can give non-real numbers for *J*, hence *C*. For this reason, we develop the optimization method in the next section.

#### Case of zero-time-shift covariances

Before presenting the new optimization, we show that *J* cannot be fully recovered from the zero-shift covariance ${\hat{Q}}^{0}$ alone. We use similar calculations to those presented in previous work [27] to evaluate the solution for *Q*^0^ in Eq (6) when *J* is diagonalizable and Σ is known. Because ${\hat{Q}}^{0}$ is symmetric, it is diagonalizable in an orthogonal basis: ${\hat{Q}}^{0}=P_{Q}D_{Q}P_{Q}^{†}$ with *D*_*Q*_ diagonal and the unitary matrix *P*_*Q*_ such that $P_{Q}^{-1}=P_{Q}^{†}$. Reorganizing Eq (6), we obtain

$$\left[P_{Q}^{†}\;J\;P_{Q}\right]D_{Q}+D_{Q}{\left[P_{Q}^{†}\;J^{†}\;P_{Q}\right]}^{†}=-P_{Q}^{†}\Sigma P_{Q}\;.$$ (10)

The solution *J* is thus given by

$$\begin{array}{rr} J & =P_{Q}FP_{Q}^{†},\\ \mu_{j}F_{ij}+\mu_{i}F_{ji} & =-G_{ij}\;,\\ G & =P_{Q}^{†}\Sigma P_{Q} \end{array}$$ (11)

with *μ*_*i*_ the real eigenvalues of *D*_*Q*_ on its diagonal. The second equality in Eq (11) implies that there are infinitely many solutions for *J*: for each pair *i* ≠ *j*, there is one degree of freedom in determining *F*, hence *J*. In other words, ${\hat{Q}}^{0}$ alone does *not* carry sufficient information to recover the whole *J* and *C*. There is, however, a unique particular solution for *F* that is symmetric:

$$F_{ij}=F_{ji}=-\frac{G_{ij}}{\mu_{i}+\mu_{j}}\;,$$ (12)

corresponding to a unique symmetric *J*. Recall that this solution requires knowledge about the noise matrix Σ.

### Lyapunov optimization (LO) to estimate network connectivity from empirical covariances

As will be shown later in Results, the direct method does not work well with noisy empirical ${\hat{Q}}^{0/\tau }$. We thus propose an alternative estimation method, where *C* is tuned iteratively such that the model reproduces the covariance observables, as illustrated in Fig 1H. Considering the noise matrix Σ to be known for now, we optimize *C* in order to reduce the model error

$$V\left(C\right)=\sum_{m,n}{\left(Q_{mn}^{0}-{\hat{Q}}_{mn}^{0}\right)}^{2}+\sum_{m,n}{\left(Q_{mn}^{\tau }-{\hat{Q}}_{mn}^{\tau }\right)}^{2}\;,$$ (13)

for a pair of objective covariance matrices ${\hat{Q}}^{0}$ and ${\hat{Q}}^{\tau }$ with a given *τ* > 0; in Results, it is referred to as *τ*_*est*_. Being the sum of two matrix distances, the Lyapunov function *V* is positive definite and becomes zero only when the two covariance matrices are equal to the objective counterparts. Eq (8) ensures the unicity of the connectivity *C* for a given pair of covariances matrices, when the solution exists.

Starting from zero weights initially, each optimization step aims to reduce *V*. To do so, we calculate the Jacobian *J* in Eq (5) for the current connectivity *C*, then the model matrices *Q*^0^ and *Q*^*τ*^ using Eqs (6) and (7) as we know Σ. We want to update *C* to obtain the following desired changes for the model covariances *Q*^0^ and *Q*^*τ*^:

$$\begin{array}{ll} \Delta Q_{mn}^{0} & =\epsilon_{C}({\hat{Q}}_{mn}^{0}-Q_{mn}^{0})\;,\\ \Delta Q_{mn}^{\tau } & =\epsilon_{C}({\hat{Q}}_{mn}^{\tau }-Q_{mn}^{\tau })\;, \end{array}$$ (14)

where the optimization rate *ϵ*_*C*_ is a some small positive number. To evaluate the Jacobian update Δ*J* that corresponds to Δ*Q*^0^ and Δ*Q*^*τ*^, we consider the equivalent of Eq (8) for the theoretical covariance matrices, namely *J* = **logm**(*X*)^†^/*τ* with *X* = (*Q*^0^)^−1^ *Q*^*τ*^. We perform an implicit differentiation with respect to *J* and *X*, yielding

$$\begin{array}{ll} \Delta J & =\frac{1}{\tau }{\left(\Delta X\;X^{-1}\right)}^{†}\\ & =\frac{1}{\tau }\;{\left\{\left[{\left(Q^{0}\right)}^{-1}\Delta Q^{0}{\left(Q^{0}\right)}^{-1}Q^{\tau }+{\left(Q^{0}\right)}^{-1}\Delta Q^{\tau }\right]\;{\left[{\left(Q^{0}\right)}^{-1}Q^{\tau }\right]}^{-1}\right\}}^{†}\\ & =\frac{1}{\tau }\;{\left[{\left(Q^{0}\right)}^{-1}\;\left(\Delta Q^{0}+\Delta Q^{\tau }expm(-J^{†}\tau )\right)\right]}^{†}\;. \end{array}$$ (15)

Finally, the desired connectivity update is simply for all *i* ≠ *j*

$$\Delta C_{ij}=\Delta J_{ij}\;,$$ (16)

and zero for the diagonal elements.

To obtain the rhs of the first line in Eq (15), we have assumed that matrices *X* and Δ*X* commute. For the LO procedure, this implies the existence of a path of matrix increments corresponding to Δ*J* such that the commutation requirement is satisfied at each step, in order to reach the global optimum. Assuming *X* to be arbitrary, the subspace of commuting matrices in which Δ*X* is constrained is a linear subspace of dimension *n*(*n*−1)/2 with *n* the number of nodes, to be compared to the dimension *n*^2^ of the space of *X*. Although this subspace is not dense, it is sufficiently rich to hope that suitable paths exist and the optimum can be approached; this will be verified using numerical simulation.

This optimization process may also not reach the global optimum when ${\hat{Q}}^{\tau }$ and ${\hat{Q}}^{0}$ do not correspond to a real solution for *C*, such as noisy empirical covariances. We will thus verify the estimation performance numerically, using the known theoretical covariance matrices obtained from Eqs (6) and (7) to check for a given connectivity matrix *C*.

#### LO estimation of a symmetric network connectivity based on zero-time-shifted covariances only

Here we show how to perform a similar iterative optimization based on ${\hat{Q}}^{0}$ only as an objective, as was done for the direct calculation method. Namely, a symmetric Δ*J* can be calculated in terms of $\Delta Q^{0}=\epsilon_{C}\left({\hat{Q}}^{0}-Q^{0}\right)$ at each optimization step. The desired Jacobian update for *J* can be written as $\Delta J=\sum_{m,n}\Delta Q_{mn}^{0}\frac{\partial J}{\partial Q_{mn}^{0}}$. Using similar calculations to those following Eq (10), we differentiate Eq (6) with respect to *Q*^0^ to evaluate $\partial J/\partial Q_{mn}^{0}$:

$$\frac{\partial J}{\partial Q_{mn}^{0}}Q^{0}+J\left(E^{mn}+E^{nm}\right)+Q^{0}\frac{\partial J^{†}}{\partial Q_{mn}^{0}}+\left(E^{mn}+E^{nm}\right)J^{†}=0\;,$$ (17)

where *E*^*mn*^ is the matrix filled with zero except for a 1 at indices *m*, *n*. Note that, because *Q*^0^ is symmetric, $\partial Q^{0}/\partial Q_{mn}^{0}=E^{mn}+E^{nm}$ and not *E*^*mn*^ alone. This binds linearly the elements ∂*J*/∂*Q*_*mn*_ and *E*^*mn*^+*E*^*nm*^, which actually leads to an equality for Δ*J* that has the same form as Eq (6):

$$\Delta J\;Q^{0}+Q^{0}\;\Delta J^{†}+J\;\Delta Q^{0}+\Delta Q^{0}\;J^{†}=0\;.$$ (18)

We have a similar indetermination as before, which can be lifted by choosing the particular symmetric solution for Δ*J*:

$$\begin{array}{rr} \Delta J & =P_{Q}KP_{Q}^{†}\;,\\ K_{ij} & =-\frac{L_{ij}}{\mu_{i}+\mu_{j}}\;,\\ L & =P_{Q}^{†}\left[J\;\Delta Q^{0}+\Delta Q^{0}\;J^{†}\right]P_{Q}\;, \end{array}$$ (19)

where the diagonal matrix *D*_*Q*_ with eigenvalues *μ*_*i*_ and the passage matrix *P*_*Q*_ are the same as defined above in Eq (10). Note that we do not need to assume knowledge about Σ here.

### Tuning of input noise variances

So far, we have considered Σ to be known and fixed during the optimization. Now we extend the theory to optimize Σ in parallel with *C*. We focus on the case where Σ is diagonal with elements Σ_*ii*_ = (*σ*_*i*_)^2^ that are the variances of the noise experienced by each network node. The key to adjust each Σ_*ii*_ is to reproduce the corresponding diagonal element ${\hat{Q}}_{ii}^{0}$. To do so, the Σ_*ii*_ are simply modified at each optimization step based on the difference between the variance of the *i*^*th*^ node of the model and its objective counterpart:

$$\Delta \Sigma_{ii}=\epsilon_{\Sigma }\left({\hat{Q}}_{ii}^{0}-Q_{ii}^{0}\right)\;,$$ (20)

where the update rate is *ϵ*_Σ_.

The implications of tuning the noise matrix Σ will be explored in a dedicated section of Results. Intuitively, it can be seen from Eq (6) that Σ affects the model covariance *Q*^0^, hence *Q*^*τ*^ via Eq (7); see Methods for reference. Therefore, assuming an erroneous Σ for the model implies incorrect desired updates Δ*Q*^0^ and Δ*Q*^*τ*^, leading to a wrong update Δ*C*.

### Heuristic optimization procedure for network connectivity

We also compare the LO method with a heuristic optimization, where existing weights are increased or decreased depending on the *Q*^*τ*^ difference between the model and objective for the corresponding matrix element for a given *τ*

$$\Delta C_{ij}=\epsilon_{\text{heur}}\left({\hat{Q}}_{ij}^{0}-Q_{ij}^{0}+{\hat{Q}}_{ij}^{\tau }-Q_{ij}^{\tau }\right)\;.$$ (21)

This method used previously [12] is adapted here to spatio-temporal covariances.

### Network configuration and model parameters used in numerical simulation

Unless stated otherwise, we use the parameters in Table 2 and Euler’s approach to simulate Eq (3) numerically. The code is written in python using the packages numpy and scipy, which incorporate a version of the Bartels-Stewart algorithm. The topology for the cluster-hub networks as in Fig 1A consists of two groups of 30% and 60% of the *N* nodes. Each group is connected recurrently with probability *p*_*con*_, with a weight randomly chosen in [10, 100]% of *c*_*max*_. We exclude self connections, although this is not crucial. The two groups are connected via hubs with a higher probability equal to 1.3*p*_*con*_ and a weight in the same range as other connections. Hubs do not have connections between each other. For random networks, all pairs of nodes have the probability *p*_*con*_ to be connected, with a random weight in [10, 100]% of *c*_*max*_. Simulations of duration *T* with identical parameters but distinct initial conditions are repeated *n*_*sim*_ times in order to evaluate the empirical *Q*.

**Table 2:** Table of simulation, network and optimization parameters for the ND artificial network model.

simulation duration	*T* = 300 s
simulation timestep	50 ms
simulation number	*n*_*sim*_ = 50
network size	*N* = 50
probability of connection	*p*_*con*_ = 20%
maximum weight for *C*	*c*_*max*_ = 0.2
time constant for neural dynamics	*τ*_*x*_ = 1 s
homogeneous input	*e* = 0.3
noise variance	$\Sigma_{ii}=\sigma_{i}^{2}=0.6$
*C* optimization rate	*ϵ*_*C*_ = 2 × 10^−4^
Σ optimization rate	*ϵ*_Σ_ = 10^−1^

Unless stated otherwise, these parameters are used for artificial networks.

We also compare our ND model with the DMF model [27] that simulates a network of neural population with AMPA and NMDA conductances, whose synaptic activity determines the BOLD signal via a hemodynamic nonlinear filter [29]. The equations regulating the synaptic activity variable *S*_*i*_ with 1 ≤ *i* ≤ *N* for the DMF are

$$\begin{array}{ll} \frac{\text{d}S_{i}}{\text{d}t} & =\frac{-S_{i}}{\tau_{NMDA}}+\beta (1-S_{i})H(u_{i})\\ u_{i} & =J\left(w_{EE}S_{i}+G\underset{k}{\sum }C_{ik}S_{k}\right)+I_{0}\\ H(x) & =\frac{ax+b}{1+\text{exp}[-c(ax+b)]}\;. \end{array}$$ (22)

The HRF model relies on the synaptic activity *S*_*i*_ to calculate the intermediate variables *s*_*i*_, *f*_*i*_, *ν*_*i*_ and *q*_*i*_ for each area, which give the BOLD signal *B*_*i*_:

$$\begin{array}{ll} \frac{\text{d}s_{i}}{\text{d}t} & =S_{i}-\kappa s_{i}-\gamma (f_{i}-1)\\ \frac{\text{d}f_{i}}{\text{d}t} & =s_{i}\\ \frac{\text{d}\nu_{i}}{\text{d}t} & =\frac{f_{i}-\nu_{i}^{1/\alpha }}{\tau_{\text{H}}}\\ \frac{\text{d}q_{i}}{\text{d}t} & =\frac{1}{\tau_{\text{H}}}\left[\frac{f_{i}}{\rho }[1-{\left(1-\rho \right)}^{1/f_{i}}]-q_{i}\nu_{i}^{1/\alpha -1}\right]\\ B_{i} & =V_{0}*[7\rho (1-q_{i})+2(1-q_{i}/\nu_{i})+(2\rho -0.2)(1-\nu_{i})]\;. \end{array}$$ (23)

All parameters are recapitulated in Table 3.

**Table 3:** Parameters for the dynamic mean-field (DMF) model and hemodynamic response function (HRF).

*a* = 270
*b* = 108
*c* = 0.154
*β* = 0.641
*J* = 0.261
*w*_*EE*_ = 0.9
*G* = 2.5
*κ* = 0.65
*γ* = 0.41
*τ*_*H*_ = 0.98
*α* = 0.32
*ρ* = 0.34
*V*_0_ = 0.02

All parameters are taken from previous publications [27, 29]. The weights of the *C* matrices are chosen in the same range for both ND and DMF models. The value for *G* sets the regime of the DMF close to the bifurcation [27].

### Experimental data set

Resting-state BOLD signal time series and dwMRI data were acquired for 25 healthy subjects aged between 18 and 39 years (mean 26.7), including 12 females and 13 males. A detailed description of the data acquisition can be found in [35], which we briefly summarize here. Subjects were asked to stay awake and keep their eyes closed, during which joint MRI-EEG recordings were performed using a 3 Tesla Siemens Trim Trio MR scanner and a 12-channel Siemens head coil [36, 37]. Acquired time series are spatially aggregated for voxels corresponding to the same area according to the parcellation. Only fMRI data are used in the present study: the BOLD signals were recorded for about 20 minutes (661 time points taken every 2 s). The BOLD signals are high-passed filtered above 0.01 Hz. Then the FC matrices correspond to covariances between all 68 regions, which are calculated using Eq (2) taking each of the 25 subjects as a separate session. Time shifts are multiples of the temporal resolution after preprocessing (2 s). For the most part, we use for FC the average covariance matrix over all subjects; a comparison with optimization based on FC for individual subjects is also presented in ‘Robustness of LO applied to experimental data. For each participant, dwMRI was used to evaluate the white-matter intracortical connectivity. Tractography is performed using corrections for motion, eddy currents, crossing pathways and the size of regions. Processing steps for MRI data include 1) preprocessing of T1-weighted scans, cortical reconstruction, tessellation and parcellation, 2) transformation of anatomical masks to diffusion space, 3) processing of diffusion data, 4) transformation of anatomical masks to fMRI space, 5) Processing of fMRI data. The dwMRI matrix used in the application to experimental data is the average over all subjects, which is thresholded to determine weights to tune in the optimization.

### Ethics statement

The paper makes a secondary use of experimental data already published [35]. The experiments have been approved by the Ethic Committee of Charite University (Berlin).

## Results

The goal of the present study is to tune a dynamic model of cortical activity to reproduce empirical FC obtained from fMRI data. Here FC corresponds to the spatio-temporal covariances ${\hat{Q}}^{0/\tau }$. The optimized network parameters are the matrices of recurrent connectivity *C* and noise Σ, the latter being diagonal. We perform the Lyapunov optimization (LO) developed in Methods to find the parameters that minimize the error between two model *Q*^0/*τ*^ matrices and their empirical counterparts ${\hat{Q}}^{0/\tau }$, as illustrated in Fig 1H. The application to fMRI data is presented in the last two subsections of Results, where the tuned *C* is an estimate of directed intracortical EC. In particular, the asymmetry of EC relates to the non-reciprocality of cortical interactions and to the difference between the strengths of incoming and outgoing connections for each cortical area.

The first part of Results concerns artificial network models, on which we test the LO procedure. We examine the influence of the choice for the time shift *τ*_*est*_ related to the objective ${\hat{Q}}^{\tau }$ upon the performance. We also show that it is necessary to tune the noise Σ received by the network nodes in order to accurately estimate the recurrent connectivity *C*. Our LO procedure is compared to two alternative to estimate the strengths of individual connections in a network, namely the ‘direct’ and ‘heuristic’ methods. Before applying our method to experimental data, we also investigate the mapping between EC and FC in a network model equipped with hemodynamics to generate the BOLD signal from neural activity. Specifically, we verify that time-shifted BOLD covariances convey information about the underlying connectivity between neural populations.

### Noise-diffusion (ND) with non-trivial network topology

The network model consists of interconnected neural populations that experience intrinsic noise and excite each other. Details about the mathematical formalism and model parameters are provided in Methods. For artificial networks, we mainly consider the topology in Fig 1A, where two groups of interconnected populations are linked via hubs. An example of the connectivity matrix is displayed in Fig 1B, where the matrix element indexed by *ij* corresponds to the connection from area *j* to *i*. Although hubs (cyan) are connected to both groups and not between each other (as indicated red arrow), they seem to make one with the large group (light green) in *Q*^0^ and *Q*^*τ*^ (see the blue arrow in Fig 1D). In contrast, the small group (dark green in Fig 1A and 1B) does not appear clearly as it exhibits rather low covariances. The reason behind this choice is to check whether the method can recover the correct topology from the *Q*^0/*τ*^ values that significantly deviate from the underlying connectivity strengths in *C*.

Our estimation method uses the spatio-temporal information in the covariance $\hat{Q}$ of the entire network to recover the directed weights in *C*, implicitly relying on the asymmetric matrix ${\hat{Q}}^{\tau }$ for *τ* > 0. In general, *Q*^*τ*^ is not symmetric for *τ* > 0 for an arbitrary *C*: Fig 1F shows that the asymmetry of *Q*^*τ*^ linearly scales on average with that of *C*, with a slope that increases with *τ*. We measure the asymmetry using the following index

$${\text{asym}}_{M}=0.5\frac{\sum_{i\neq j}\left|M_{ij}-M_{ji}\right|}{\sum_{i\neq j}\left|M_{ij}\right|}$$ (24)

for a matrix *M*. It ranges from 0 for symmetric matrices to 1 for antisymmetric matrices. Large weights with no or weak reciprocal connection mainly contribute to the index. The relationship in Fig 1F can be understood looking at the expression for *Q*^*τ*^ in Eq (7), which amounts to *Q*^0^ multiplied by the matrix exponential **expm**(*J*^†^ *τ*). The Jacobian *J*^†^ is the transpose of the Jacobian matrix defined in Eq (5) and has the same off-diagonal elements as *C*, so asym_*J*_ = asym_*C*_. For small *τ*, *Q*^*τ*^ can be approximated by *Q*^0^ + *Q*^0^ *J*^†^ *τ*, which explains why asym_*Q*^*τ*^_ is smaller than asym_*C*_. The variability of the *Q*^*τ*^ asymmetry compared to the small slopes of the curves makes it difficult to infer the asymmetry of *C*. This further motivates our method to estimate accurately *C* from *Q*.

We focus on the case where the recurrent weights are sufficiently large to generate a strong network effect: the mapping between *C* and *Q* is not trivially linear and many large values in *Q*^0^ correspond to nodes that are only weakly or not connected. Here, hubs in the network in Fig 1A are not connected, but have strong variances in Fig 1G and covariances between each other due to their numerous connections.

### Test of Lyapunov optimization (LO) on artificial networks

The optimization procedure is summarized in Fig 1H: *C* is iteratively modified such that the model covariance matrices *Q*^0^ and *Q*^*τ*^ converge towards the objective covariance matrices ${\hat{Q}}^{0}$ and ${\hat{Q}}^{\tau }$, for a chosen *τ* = *τ*_*est*_ > 0. In the first place we assume Σ to be known. Starting from a initial matrix *C* with all weights equal to zero, LO calculate a *C* update at each step such that it reduces the Lyapunov function *V* in Eq (13); *V* is the sum of the matrix distance between *Q*^0^ and ${\hat{Q}}^{0}$ on the one hand, and that between *Q*^*τ*^ and ${\hat{Q}}^{\tau }$ on the other hand. To measure the goodness of fit of *C* or *Q*, we define the model ‘error’ as the normalized distance for a matrix *M* and its objective *M*^*obj*^:

$$d\left(M,M^{\text{obj}}\right):=\frac{\sum_{\left(i,j\right)}{\left(M_{ij}-M_{ij}^{\text{obj}}\right)}^{2}}{\sum_{\left(i,j\right)}{\left(M_{ij}^{\text{obj}}\right)}^{2}}\;,$$ (25)

which are involved in *V*.

**Fig 2:**
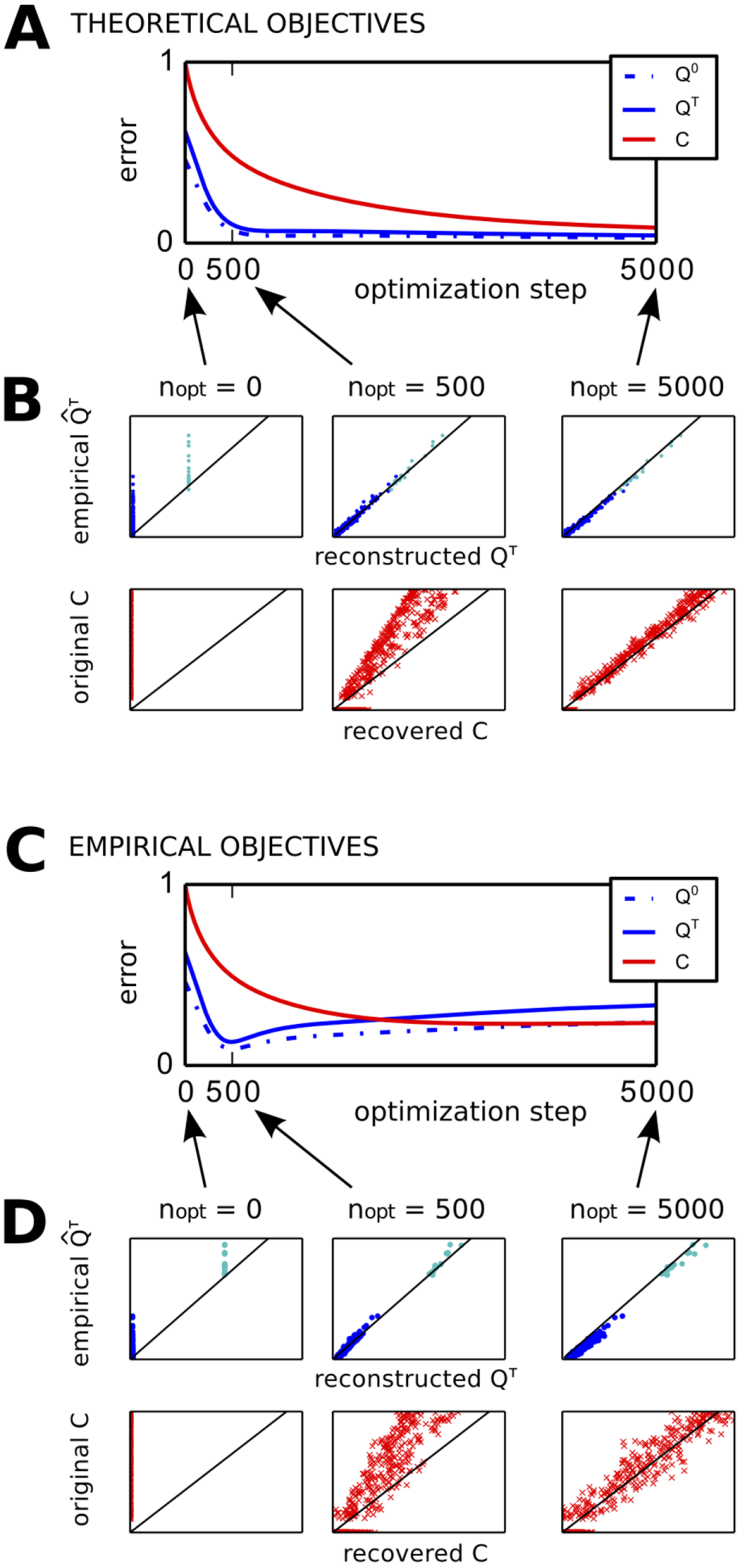
Iterative optimization to recover *C* from empirical *Q*. **A** Estimation based on theoretical covariances. Evolution of *Q*^0^ and *Q*^*τ*^ errors between the model and theoretical objectives (blue curves), as well as *C* error between the original and model connectivity (red). The theoretical objectives ${\hat{Q}}^{0/\tau }$ are calculated using Eqs (6) and (7). Errors are calculated using the normalized matrix distance in Eq (25). The optimization relies on Eq (15) with time shift *τ*_*est*_ = 1 s. **B** Details of the match of the model and objective matrices for *Q*^*τ*^ (blue dots) and *C* (red crosses) at three stages of the optimization in A. The black line indicates a perfect match. **C** Similar plot to A with empirical *Q* as objectives, evaluated using Eq (2) for 50 simulations of the same network as in A. **D** Similar plot to B for the optimization in C.

Firstly we verify that LO recovers the correct connectivity for theoretical objectives ${\hat{Q}}^{0/\tau }$ calculated with Eqs (6) and (7) with the original *C* and Σ. A typical example for the evolution of the *C* and *Q* errors is illustrated in Fig 2A for *τ*_*est*_ = *τ*_*x*_ = 1 s. While the *Q*^0/*τ*^ errors exhibit a plateau, the *C* error keeps on decreasing. Fig 2B shows the faster convergence for *Q* than *C* for three stages of the optimization. The residual *C* error is very small; it may remain non-zero when the commutativity assumption necessary to derive Eq (15) is not satisfied.

For the same original *C*, we now simulate the network and calculate the empirical objectives ${\hat{Q}}^{0/\tau }$ using Eq (2) for 50 independent repetitions of 300 s each with timestep 0.05 s (i.e., 3 × 10^5^ data points). In Fig 2C, the *Q*^0/*τ*^ errors first drop, then rebound and stabilize. Interestingly, the *C* error continues to decrease even though the *Q* error worsens. Because of the noise in the empirical ${\hat{Q}}^{0/\tau }$, there is no real solution *C* to Eq (7) and the residual error does not vanish. In Fig 2D, the model *C* gives a better fit for the original *C* after 5000 optimization steps than for the minimum for the *Q* error. LO also allows for the use of constraints on the weights in *C*. Non-negativity is imposed here, although it does not affect the results significantly.

#### Matching time shift is crucial for accurate connectivity estimation

**Fig 3:**
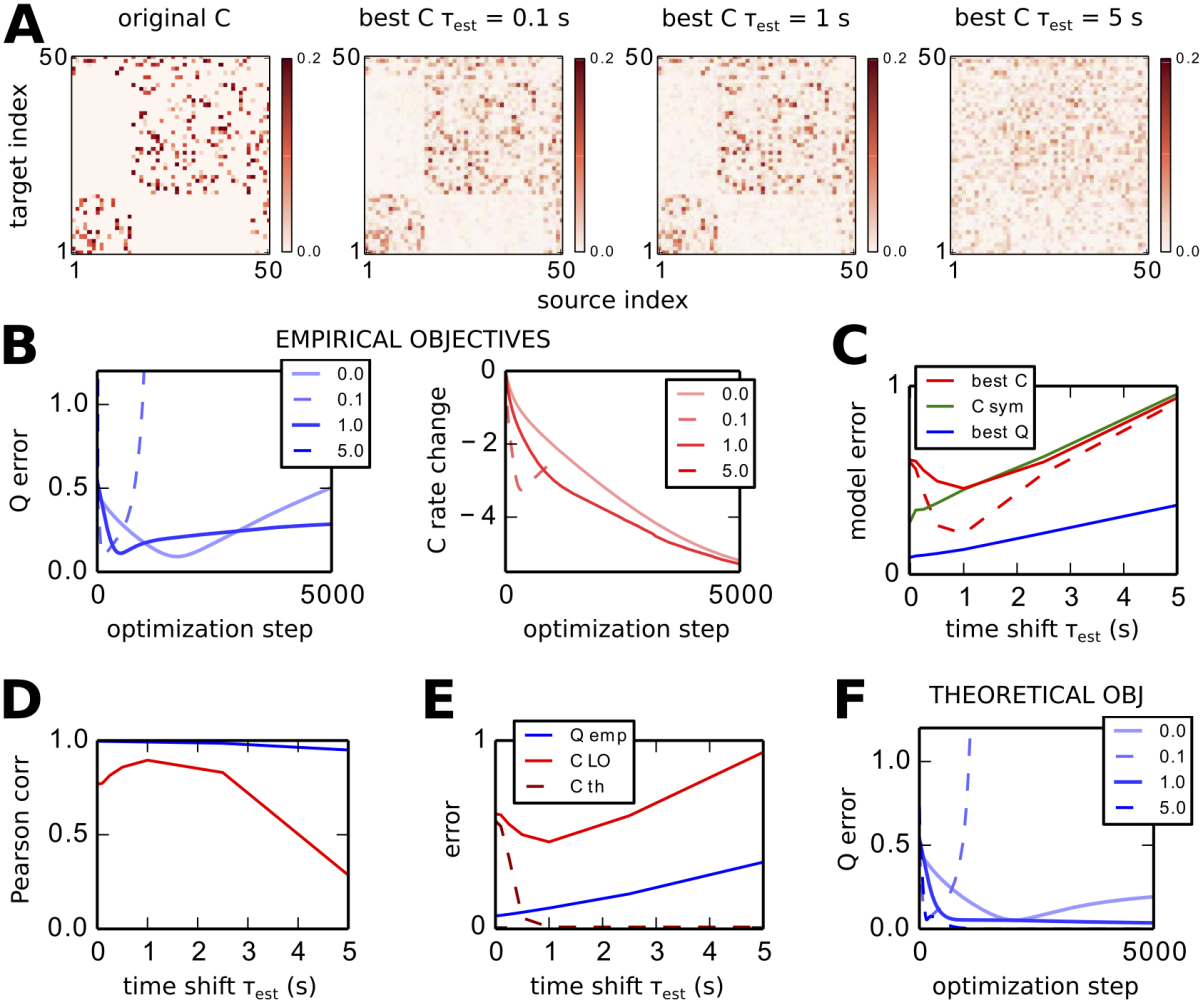
Influence of choice for *τ*_*est*_ on accuracy of recovered *C*. **A** Best estimated matrices *C* for *τ*_*est*_ = 0, 0.1, 1 and 5 s. For *τ*_*est*_ = 1, the *C* matrix at the end of the optimization (step 10000) is also shown. **B** Evolution of *Q* errors for the optimizations with various *τ*_*est*_ in A. bf C *C* errors corresponding to the best matrices in A (red solid curve), as well as last matrices of the optimization (red dashed curve). The green curve indicates the error of the best *C* with the symmetric part of the original *C*. The blue curve indicates the error between the empirical ${\hat{Q}}^{\tau }$ and the corresponding theoretical matrix. **D** Pearson correlation coefficient between the best and original *C* (red) as well as reconstructed and objective *Q*^0/*τ*^. between ${\hat{Q}}^{\tau }$ and each corresponding theoretical *Q*^*τ*^ matrix for the same *τ* = *τ*_*est*_ in C. **E** Comparison between LO with empirical $\hat{Q}$ calculated with Eq (2) (red solid curve) and LO with theoretical $\hat{Q}$ obtained from Eq (7) (red dashed curve). The blue curve indicates the inaccuracy between the empirical and theoretical *Q*^*τ*^ matrices. **F** Same as B for the *Q* error with theoretical objectives ${\hat{Q}}^{0/\tau }$.

The estimation performance depends on the choice for *τ*_*est*_ related to the empirical objectives: Fig 3A shows for several *τ*_*est*_ the ‘best’ *C*, corresponding to the minimum of the *Q* error as in Fig 2C during the optimization. From now on, the *Q* error denotes the mean normalized distance for both *Q* matrices: $(d\left(Q^{0},{\hat{Q}}^{0}\right)+d\left(Q^{\tau },{\hat{Q}}^{\tau }\right))/2$. For *τ*_*est*_ = 0.1 and 1 s, the global characteristics of the original *C* topology (left panel) are recovered, namely the two interconnected groups linked via hubs that are not connected with each other. Although weights are weaker than in the original *C*, the detail of the connectivity is recovered for *τ*_*est*_ = 1 s. In contrast, the estimated *C* for *τ*_*est*_ = 0.1 s is quasi symmetric. Last, *τ*_*est*_ = 5 s gives a poor estimation where the cluster-hub topology is hardly detected.

The left panel of Fig 3B shows the evolution of the *Q* errors for the optimizations in A. For small or large *τ*_*est*_ > 0 compared to *τ*_*x*_ = 1 s, the *Q* error often exhibits a large rebound similar to an explosion (dashed curves). LO being less stable for small and large *τ*_*est*_ > 0, it gives a poor estimate for *C*. In the case *τ*_*est*_ = 0, the optimization using Eq (19) is rather stable; however, the resulting estimate is closer to the symmetric part of the original *C*, not the directed matrix. The rate of change for *C* is displayed in Fig 3B (right): during the rebound or plateau of the *Q* error, the *C* matrix stabilizes for *τ*_*est*_ = 0 and 1 s (solid curves).

Fig 3C recapitulates our first main theoretical finding: *τ*_*est*_ needs to be matched with *τ*_*x*_ = 1 s in order to obtain an accurate estimation (see *C* errors in red). The dashed red curve corresponds to the *C* for the minimum of the rate of change (red curves in Fig 3B), yielding a better *C* recovery than the solid red curve that corresponds to the minimum of the *Q* error (blue curves). The green curve indicates the matrix distance to the symmetric part of the original *C*, namely 0.5(*C*+*C*^†^). It shows that LO for small *τ*_*est*_ > 0 recovers the undirected connectivity like *τ*_*est*_ = 0, rather than the correct directed one. Finally, we observe that the best *Q* error corresponding to the minimum over the optimization (blue curve) is not a good indicator for the *C* error (red curve): it does not provide useful insight for choosing *τ*_*est*_. The performance is further illustrated in Fig 3D by the Pearson correlation coefficient between the best and empirical *C* (red curve) and *Q*. The best *C* for *τ*_*est*_ = 1 s corresponds to values larger than 0.8, which means that the weight ranking of the original *C* is well captured.

In Fig 3E, we compare LO for empirical objectives (red solid curve) with LO for theoretical objectives (red dashed curve). For *τ*_*est*_ > 0, even theoretical objectives give a large *C* error, meaning that the poor performance comes from the procedure itself. This can be intuited from Eq (15), which relies on a “contrast” between ${\hat{Q}}^{0}$ and ${\hat{Q}}^{\tau }$: for small *τ*_*est*_, these two matrices are too similar and the *C* update is subject to numerical imprecision. This is confirmed by the evolution of the *Q* error in Fig 3F. For large *τ*_*est*_, however, theoretical objectives yield a very good estimation. This means that the poor performance for large *τ*_*est*_ comes from the inaccuracy in the empirical ${\hat{Q}}^{\tau }$, as represented by the blue solid curve in Fig 3E.

#### Need for estimating intrinsic noise in addition to connectivity

**Fig 4:**
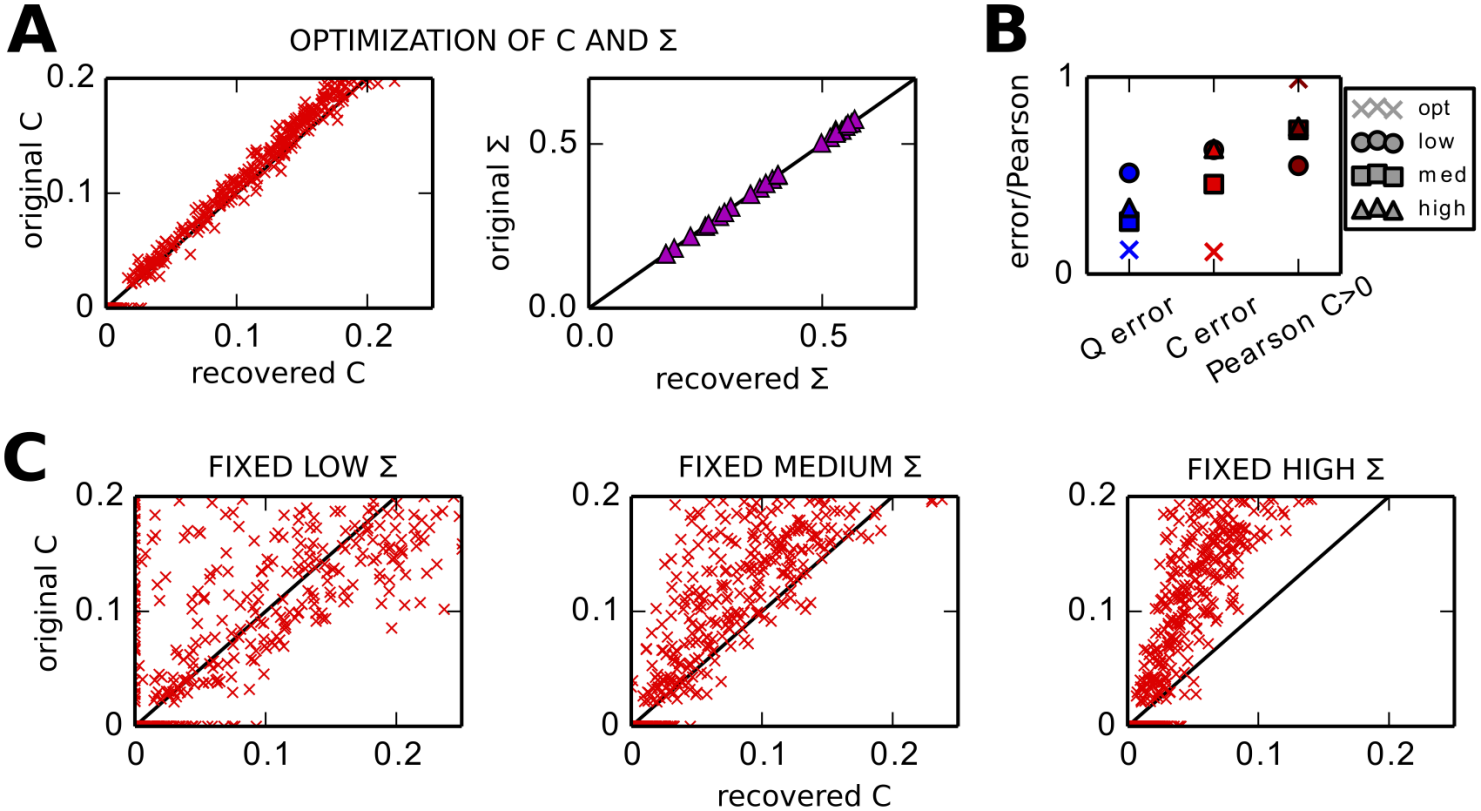
Need for tuning intrinsic noise Σ for each node in addition to *C*. **A** Match between the recovered and the original *C* (red crosses) and Σ (purple triangles) for LO based on Eqs (15) and (20) with theoretical objectives and *τ*_*est*_ = 1 s. The original network has inhomogeneous noise Σ_*ii*_. **B** Comparison of *Q* error, *C* error and Pearson correlation coefficient for non-zero weights of the original *C* for four optimizations: ‘opt’ where both *C* and Σ are tuned as in A; ‘low’ for the tuning of *C* with a fixed Σ with the minimum value of the original Σ; likewise, ‘med’ and ‘high’ with a fixed Σ with the mean and maximum value of the original Σ. **C** Match between the original and recovered *C* for the optimization with fixed homogeneous Σ for three distinct values: the minimum, mean and maximum of the values in the original Σ. They are to be compared with the left panel in A.

Now we extend the LO procedure to adjust the variance of the noise individually inputted to the network nodes: each diagonal element Σ_*ii*_ is increased or decreased such that the node variance $Q_{ii}^{0}$ of the model converges toward its objective ${\hat{Q}}_{ii}^{0}$, see Eq (20) in Methods. To verify the estimation accuracy, we consider a target network with Σ_*ii*_ elements uniformly distributed between in [0.1, 0.6] and perform LO for the theoretical objectives ${\hat{Q}}^{0}$ and ${\hat{Q}}^{\tau }$ with *τ* = 1 s obtained using Eqs (6) and (7). The match between the model and objectives for both *C* and Σ is shown in Fig 4A, corresponding to a quasi perfect estimation of the connectivity as in Fig 2B for the tuning of *C* only.

To assess the importance of tuning Σ, we perform three optimizations using a fixed homogeneous Σ for three levels of noise, namely the minimum, mean and maximum of the original Σ. As illustrated in Fig 4B, the *Q* error and, more importantly, the *C* error are larger for those cases (circle, square and triangle) than that with the tuning of Σ (crosses). Moreover, the values for the non-zero weights in the original *C* are estimated less accurately, as measured by the Pearson correlation coefficient. Fig 4C shows for each case the corresponding matches between the original and estimated *C* matrices. This is the second main theoretical finding: the optimization of Σ is as important as choosing *τ*_*est*_ to obtain an accurate estimate for *C*.

In practice, we find that a fast tuning for Σ works best in order to keep the optimization stable, i.e., avoid early “explosion” of the *Q* error. The presented simulations corresponds to *ϵ*_Σ_ = 10^−1^ in Eq (20), to be compared with *ϵ*_*C*_ = 2 × 10^−4^ to calculate Δ*Q* in Eq (14) for the *C* update, cf. Methods.

#### Benchmark of LO procedure and comparison to the direct and heuristic methods

**Fig 5:**
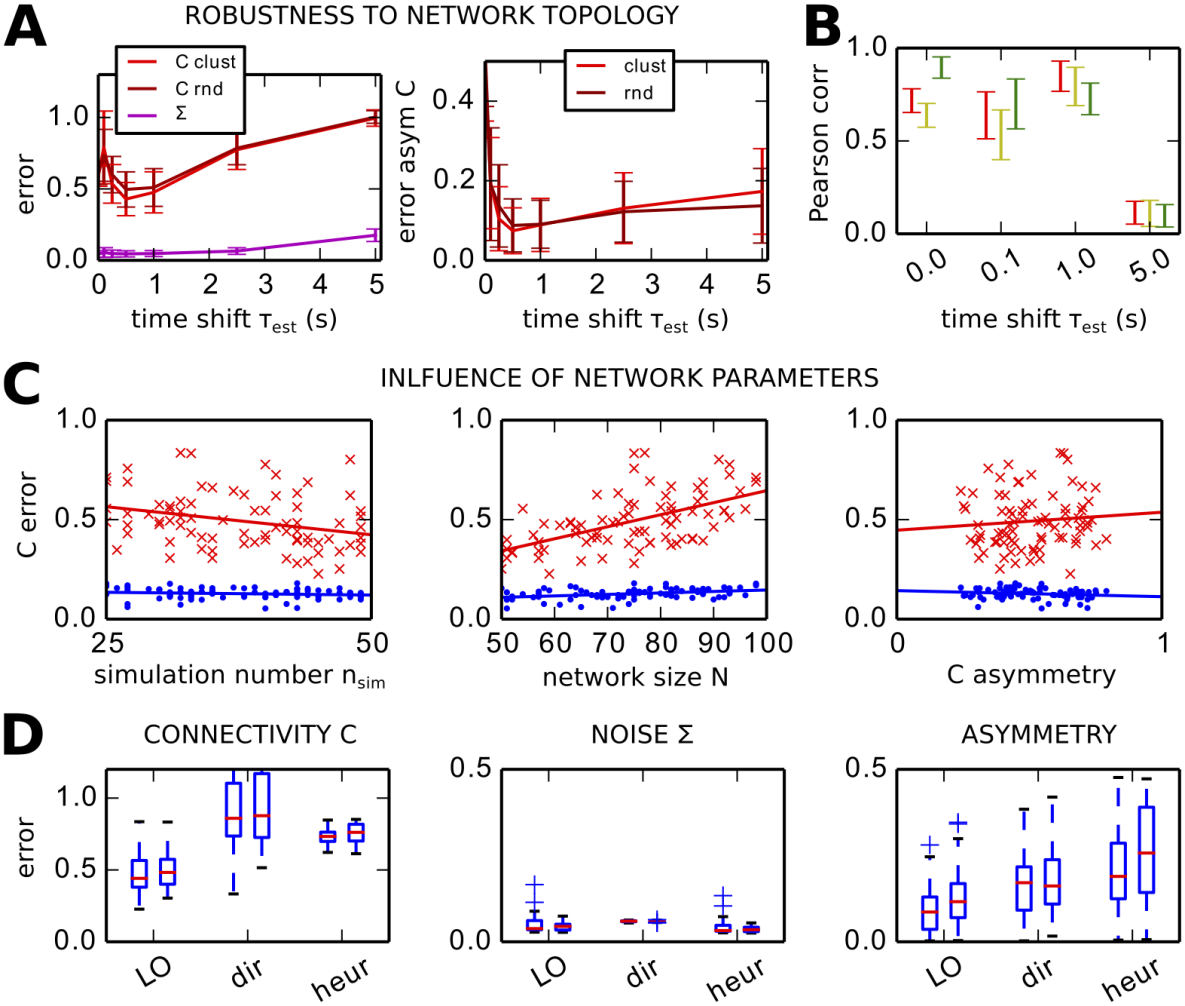
Robustness of the estimation procedure of *C* and Σ on artificial networks. **A** Left: Comparison of the *C* error (red curves) and Σ error (purple curve) for optimization based on several *τ*_*est*_. For the *C* error, the cluster-hub topology in Fig 1A corresponds to lighter red and the random connectivity to darker red. The plot is similar to Fig 3C; the error bars correspond to the standard deviation over 40 network configurations. Right: Similar plot for the error of *asym*_*C*_ for the two network topologies. **B** Pearson correlation coefficient between the matrix elements of the whole recovered and original *C* matrices in red; idem but limited to non-zero elements of the original *C* in yellow; and between the recovered *C* and the symmetric part of the original *C* in green. The plot concerns 50 networks of either topology for which the asymmetry of original *C* is larger than 0.5; LO is performed using *τ*_*est*_ = 1 s. **C** *C* and *Q* errors (in red and blue, respectively) plotted against several parameters for the network and LO: the number of simulations used to calculate the empirical $\hat{Q}$, the network size *N* and the asymmetry of the original *C*. Each cross/dot corresponds to one of the 80 simulations of either topology in A for *τ*_*est*_ = 1 s. **D** Comparison of the estimation performance of LO wit the direct and heuristic methods for the *C* error (left), the Σ error (middle) and asymmetry error (right). For each case, the left boxplot correspond to 40 cluster-hub networks and the right one to 40 randomly connected networks; the optimizations are performed with *τ*_*est*_ = 1 s. Contrary to others, the asymmetry error is not normalized.

Fig 5 combines the results found in Figs 3 and 4 to verify the robustness of LO when varying the network parameters. Fig 5A compares the *C* error for two network topologies: the lighter red curve corresponds to the cluster-hub type as in Fig 1A and the darker curve to randomly connected networks. The need for matching *τ* with *τ*_*x*_ is confirmed in the left panel for both types. The Σ error in purple is close to perfect for all *τ*_*est*_. The error bars correspond to the variability over 40 networks with randomly chosen parameters: 25 ≤ *n*_*sim*_ ≤ 50 simulations to calculate the empirical $\hat{Q}$; network size 50 ≤ *N* ≤ 100; probability of connection *p*_*con*_ ∈ [10%, 30%]; maximum recurrent weight *c*_*max*_ ∈ [1.2, 2] × 1/*N*/*p*_*con*_; input strength *e* ∈ [0.8, 1.2]; and average noise level *σ* ∈ [0.3, 0.6], with individual values shuffled ±50%; see also Table 2. No significant difference is observed there. On average over all 80 networks, LO with *τ*_*est*_ = 0.5 and 1 s give the best performance. On a technical ground, following the observations in Fig 3B and 3C, here the best *C* is chosen after the minimum for the *Q* error, provided the rate of change for *C* decreases (i.e., *C* keeps stabilizing) and the *Q* error remains smaller than 1.5 times its minimum.

The right panel of Fig 1A shows that the asymmetry of the original *C* is correctly estimated for matched *τ* and *τ*_*x*_. This means that directed connections are well estimated. Fig 5B further illustrates the quality of the estimation for the 50 networks with the strongest asymmetries: for small *τ*_*est*_ ≥ 0, the estimated *C* is closer to the symmetric part of the original *C* (largest Pearson correlations in green). For *τ*_*est*_ = 1 s, the *C* recovers well the directed connections in the original *C*, as shown by the Pearson correlations in red (all connections) and yellow (non-zero connections in the original *C*).

Fig 5C shows how LO performs depending on the amount of observed data and network parameters. As expected from previous work [25], the optimization performs better in Fig 5C when a larger number *n*_*sim*_ of simulations of the network activity is used to compute ${\hat{Q}}^{0}$ and ${\hat{Q}}^{\tau }$, see Eq (2). Connectivity in larger networks is more difficult to estimate, but the asymmetry of the original *C* does not affect much the performance.

Finally, we compare our method with the direct and heuristic methods corresponding to Eqs (8) and (21), respectively. For each case in Fig 5D, the left and right box plots correspond to the optimization with with *τ*_*est*_ = 1 s for 40 cluster-hub and 40 randomly connected networks as in Fig 5A, respectively. While the Σ error is very small for all methods, the *C* error is much smaller for LO than the others. The direct method gives large complex values in the Jacobian obtained from Eq (8) due to the “noise” in empirical ${\hat{Q}}^{0/\tau }$, which severely impairs the estimation. The heuristic method also yields a poor *C* error, although the *Q* error is very low: it does not capture the network effect correctly, namely the non-linear mapping between *C* and *Q* due to the recurrent feedback. Last, the asymmetry of the original *C* is best estimated by LO.

### Hemodynamics and temporal information in BOLD covariances

Our method relies on information in time-shifted covariances to infer the connectivity of the underlying network. In the context of cortical models of BOLD activity, EC classically denotes the connections between neural populations, whose activity is transformed into BOLD signals via a hemodynamic response function (HRF). Before applying our method to fMRI experimental data, we need to test whether BOLD time series as obtained from the HRF convey information in their time-shifted measures about EC.

**Fig 6:**
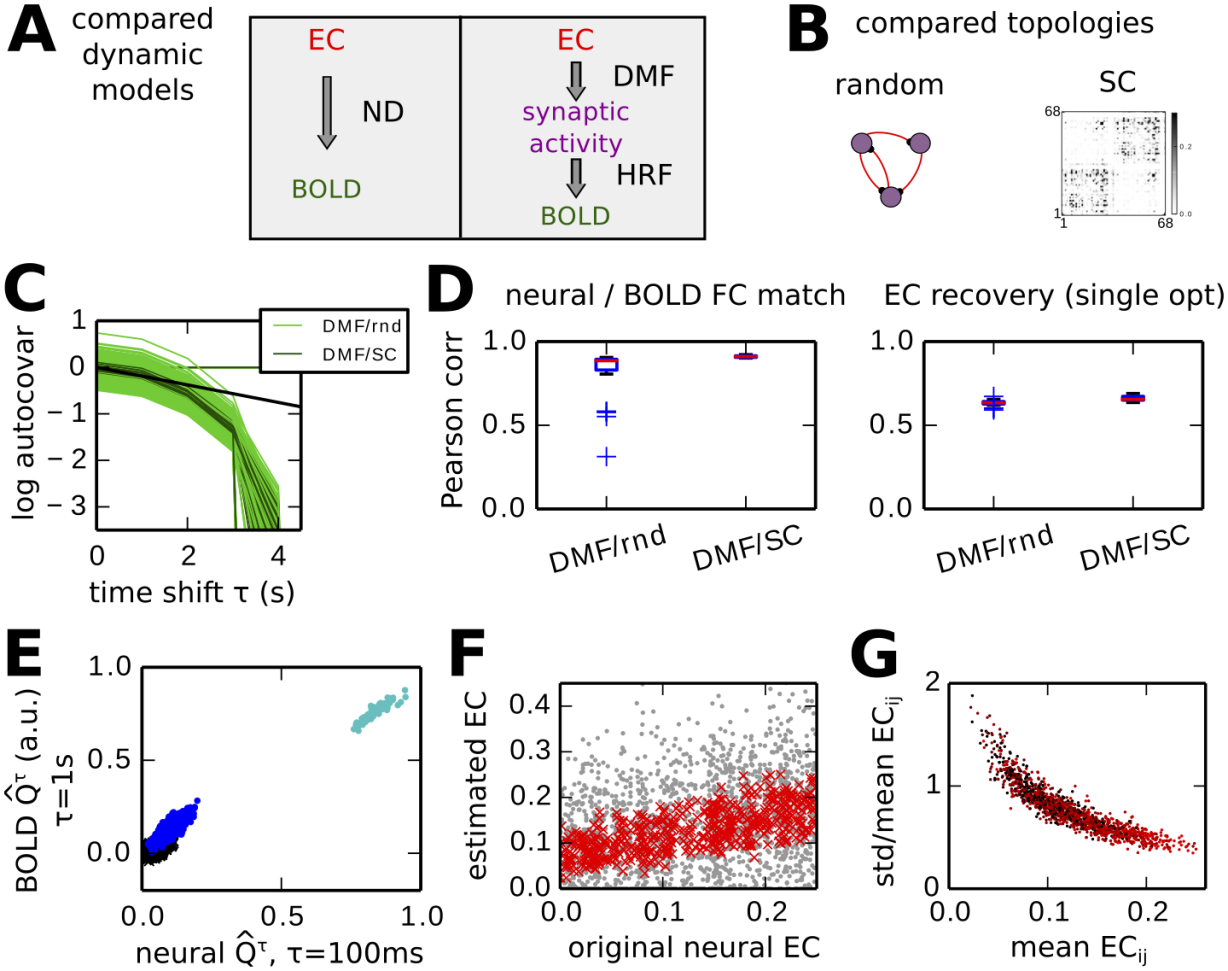
Information conveyed by spatio-temporal FC and hemodynamic response function (HRF). **A** Schematic representation of the properties of the two dynamic cortical model: ND network whose activity directly models the BOLD signals; dynamic mean-field (DMF) model whose synaptic activity is processed by the HRF to obtain the BOLD signal. **B** Two types of neural connectivity used with the DMF: random and SC matrix obtained from dwMRI. In the matrix, darker pixels indicate a higher probability of existing fibers between cortical areas. **C** Autocovariances of the two models for 50 different simulations. The y-axis has a log-scale. For each simulation, the curves are centered vertically with respect of the mean over all nodes to focus on the slope. The black line represents the exponential decay fitted on the mean experimental BOLD with time constant 5.3 s. **D** Left: Similarity between neural and BOLD covariances (excluding variances) for the two considered DMF models, as measured by the Pearson correlation coefficient. Right: Performance of EC estimation from BOLD FC for the DMF models. The performance is measured by the Pearson correlation coefficient. For each simulation, the objective is the average BOLD FC taken from 50 simulations of the same network. **E** Example of a typical mapping between neural and BOLD covariances for a DMF/SC network. The covariances have been rescaled. **F** Variability of the EC estimated from individual FCs. The grey dots represent the match of four EC each estimated for a single simulation of 300 s. The red dots correspond to the average over 50 estimated EC for 50 simulations of the same DMF/SC network. **G** Uncertainty of the estimated EC as a function of the estimated weight for each neural connection. The y-axis is the standard deviation over the 50 optimizations in F divided by the mean.

Therefore we compare our noise-diffusion (ND) network with the dynamic mean-field (DMF) model based on AMPA and NMDA dynamics [27] equipped with the Balloon/Windkessel model for the HRF [29]. As illustrated in Fig 6A, the synaptic input activity of the DMF (*u*_*i*_ in Methods) is transformed by the HRF to generate the BOLD signal. We consider the two topologies in Fig 6B: a randomly connected network and a network whose connections correspond to the SC obtained from dwMRI (see Methods). The SC connectivity has 32% density corresponding to the largest dwMRI values, as will be used in the next sections for the ND model applied to fMRI data. The model parameters are taken from previous publications [27, 29] and recapitulated in Table 3. In Fig 6C, we simulate 50 configurations of the DMF+HRF model with the two topologies (‘DMF/rnd’ and ‘DMF/SC’, respectively). For each, we choose weights randomly and calculate the empirical autocovariances of the BOLD signals. We find significant values up to time shifts equal to 3 s, which then drop. Interestingly, the slope in the log plot for the smaller time shifts is close to that found in experimental data (black line) up to 2 s of time shift. Then we estimate the similarity between the neural covariances at a short timescale and the FC given by the BOLD covariances, using the Pearson correlation coefficient between the corresponding matrix elements. As shown by the Pearson correlations in Fig 6D (left panel), the match between the neural and BOLD covariances is good, especially when EC is constrained by SC. This means that, although the HRF involves nonlinearities, the DMF model preserves the ranking of the spatio-temporal covariances is preserved between neural and BOLD activity, as illustrated in Fig 6E. In particular, the time-shifted BOLD covariance matrices are asymmetric.

Now we use the LO procedure to estimate the original EC from the BOLD FC with *τ*_*est*_ = 1 s. The right panel in Fig 6D indicates the Pearson correlation between the original and estimated connectivity. For the DMF model, the agreement corresponds to 0.6 and 0.65 for the random and SC topologies, respectively. This means a fair recovery of the ranking between the original EC weights. For the DMF/SC networks where the existing connections are “known” (32% density), LO only tunes the corresponding weights in *C*.

Finally, we find that the variability of the estimated ECs decreases with the strength of the neural connection. This suggests that strong estimated EC values can be trusted based on a threshold on the variability of the estimations (standard deviation divided by the mean). Together, these results support the applicability of our method to fMRI data and directly estimate the EC strengths from the average FC.

### Application to estimation of EC from fMRI data during rest

**Fig 7:**
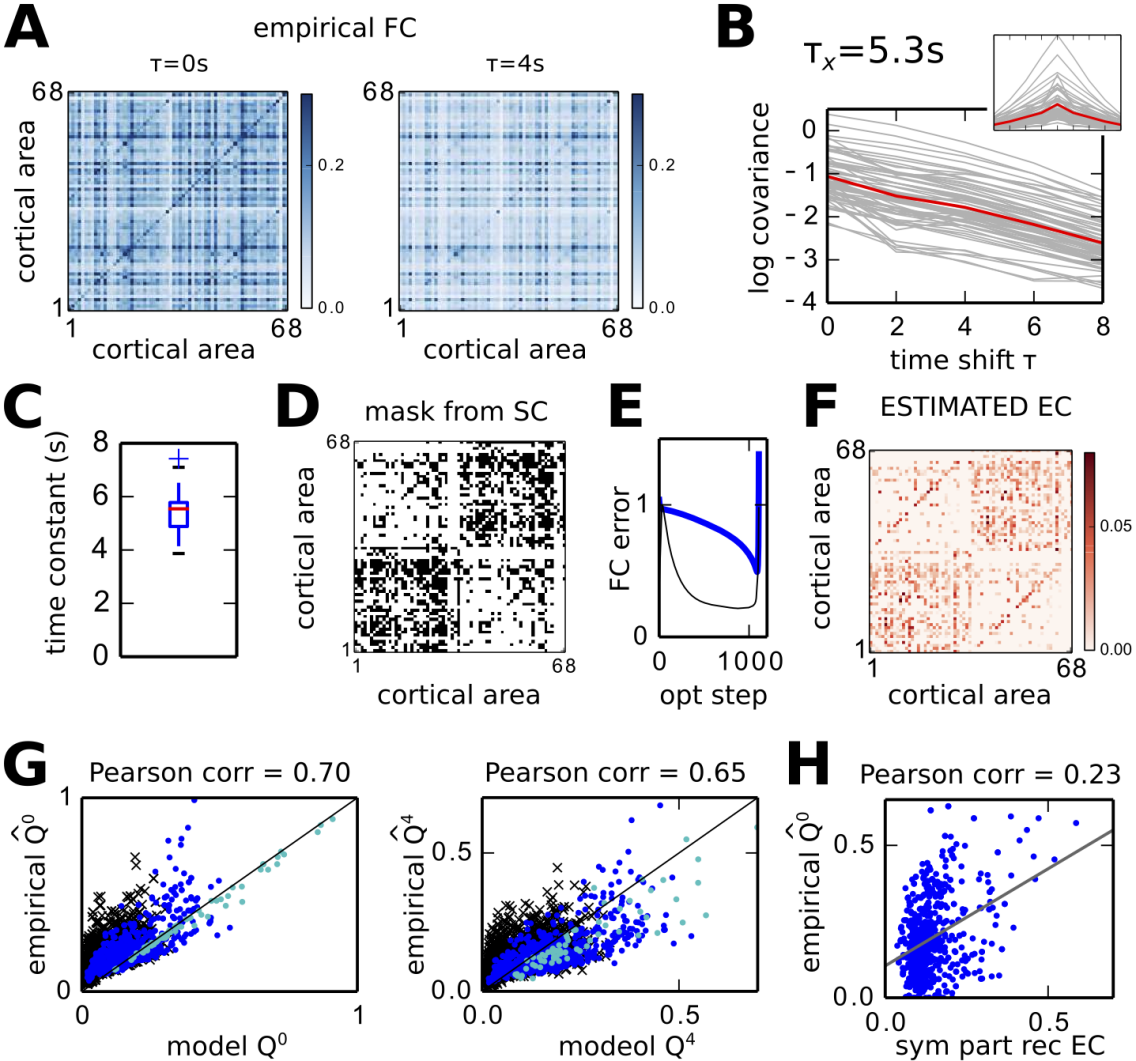
Fitting fMRI data to infer cortico-cortical connectivity. **A** Empirical FC, namely ${\hat{Q}}^{0}$ and ${\hat{Q}}^{\tau }$, averaged over 25 subjects for BOLD signal recorded during rest. **B** Autocovariances (grey curves, with a log y-axis) as a function of the time shift *τ*. From the mean curve (red) the time constant for the ND model is estimated: *τ*_*x*_ = 5.3 s. The inset represents the autocovariances with a standard y-axis. **C** Variability of similar decay time constants to *τ*_*x*_ in B for individual cortical area. **D** SC mask of weights to tune (black pixels), corresponding to the strongest connections of the dwMRI matrix in Fig 6B thresholded to obtain 32% density. **E** Evolution of the *Q* error during the optimization. The black line indicates the rate of change of *C*. **F** EC corresponding to the estimated *C* matrix. **G** Match between empirical and reconstructed *Q* matrices for the optimization with *τ*_*est*_ = 4 s while the connections for the mask in D. For both *Q*^0^ and *Q*^*τ*^, the blue dots correspond to tuned connections, the black crosses to non-diagonal untuned connections and the cyan dots to diagonal connections. The Pearson correlation coefficient between matrix elements are given above each plot. **H** Weak correlation between the symmetric part of the estimated *C* and ${\hat{Q}}^{0}$, indicating a strong network effect.

Now we use the ND model to reproduce the cortical FC obtained from fMRI recordings during rest. In contrast to previous studies [12, 27], FC involves both zero-time-shift and time-shifted covariances of the BOLD signals for the 68 cortical regions here. Following the results in Fig 6, we denote by EC the connectivity *C* in our model, even though the network activity directly models the BOLD signals without a HRF. The data set corresponds to 25 subjects aged from 20 to 39 years. The empirical covariances are calculated using Eq (2) for *τ* that are multiples of 2 s, which is the temporal resolution of the BOLD time series. Fig 7A displays the objective FC matrices ${\hat{Q}}^{0}$ and ${\hat{Q}}^{\tau }$ with *τ* = 4 s, which are averages over all subjects. The autocorrelograms of all 68 nodes are displayed in the inset of Fig 7B. The main panel shows the same curves with a log-scale for the y-axis: they seem quasi straight lines indicating exponential decays, as observed for artificial networks in Fig 1E. Therefore, we estimate from the mean BOLD autocovariance over all nodes (red curve of Fig 7B) the time constant *τ*_*x*_ = 5.3 s, which we use to calibrate the model. We use a single *τ*_*x*_ for all nodes, because the distribution of individual time constants over all nodes is narrow, as shown in Fig 7C.

The direct method in Eq (8) does not work here: the reason is that the matrix logarithm gives complex values with large imaginary parts. This motivated the development of the LO procedure. Moreover, our approach allows for the use of constraints on the weights in *C*: only a subset of all possible cortico-cortical connections can be tuned. The mask in Fig 7D represents the anatomical cortical connectome obtained from thresholding the dwMRI data averaged over all subjects: connections with large SC values are optimized, other weights are kept equal to zero. The density of existing connections is 32% in Fig 7D.

The blue curve in Fig 7E represents the evolution of the *Q* error during LO with *τ*_*est*_ = 4 s: it first decreases and then “explodes”. This instability corresponds to a transition to excessively high activity due to too strong feedback. The black curve show the normalized rate of change for *C*, whose minimum is close to that of the *Q* error. For the experimental data, the minimum of the black curve comes before the minimum of the *Q* error, but the rate of change of *C* remains close to its minimum until the explosion. In the following, we choose the estimated EC in Fig 7F and reconstructed FC as the *Q* and *C* for the minimum of the *Q* error. The match between the model and empirical *Q* matrices is illustrated in Fig 7G. Importantly, the match is similar for the *Q* corresponding to tuned connections (blue dots) and absent connections (black crosses). The fit of variances corresponding to the diagonal elements of *Q* (cyan dots) is very good.

Fig 7H indicates the poor match between the empirical FC and the symmetric part of estimated *C*: large values in ${\hat{Q}}^{0}$ arise from strong network effect even though the EC weight is low.

#### Robustness of LO applied to experimental data

**Fig 8:**
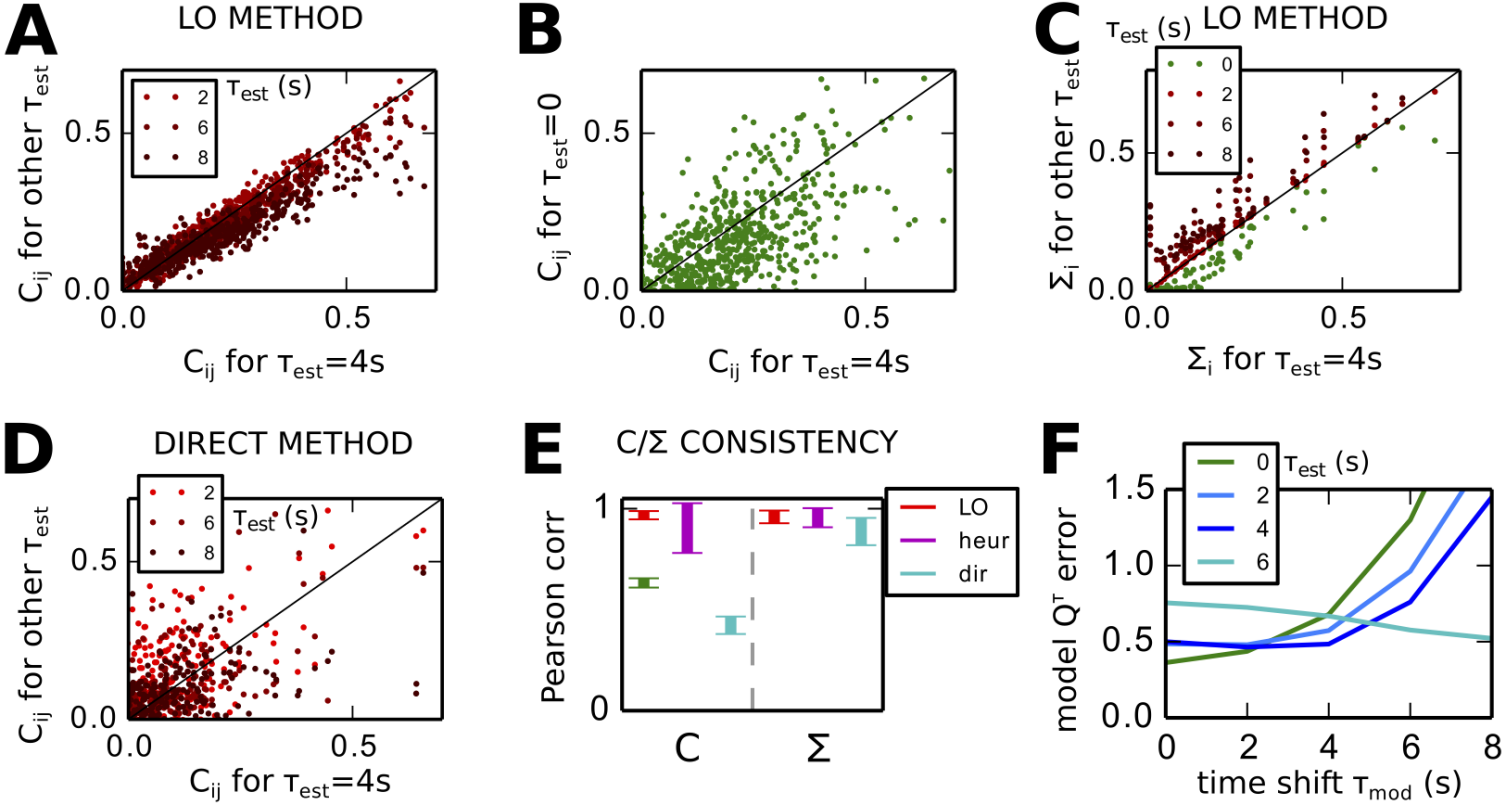
Robustness of estimated *C* and Σ from FC with different time shifts *τ*_*est*_. **A** Match between *C* for *τ*_*est*_ = 4 s (x-axis) and *C* for *τ*_*est*_ = 2, 6 and 8 s (y-axis). Each plotted point corresponds to an EC connection and the black line indicates a perfect match. **B** Same as B for the EC estimated for *τ*_*est*_ = 0 on the y-axis. **C** Similar plot to A for the diagonal elements of the estimated noise matrix Σ. **D** Similar plot to *B* for the EC estimated using the direct method. **E** Agreement as measured by the Pearson correlation coefficient between the pairs of EC and Σ plotted in B and D for the LO (red), heuristic (purple) and direct (cyan) methods. The green error bar on the left represents the same between EC with *τ*_*est*_ = 0 and the four *τ*_*est*_ > 0. **F** Comparison of the models tuned with LO using different time shifts. For each model *C* obtained with *τ*_*est*_ from 0 to 6 s, the curve represents the *Q*^*τ*^ error for all *τ*_*mod*_ on the x-axis.

Before analyzing the properties of the estimated EC, we check the consistency of the *C* and Σ estimated by LO. As shown in Fig 8A, the best *C* matrices for *τ*_*est*_ ≥ 2 s describe a similar network topology, i.e., approximately the same ranking between weights (the black line indicates a perfect match). In contrast, the best *C* obtained from *Q*^0^ only does not match the best *C* for *Q*^0^ and *Q*^4^, as illustrated in Fig 8B. The estimated intrinsic noise Σ in Fig 8C is similar for all *τ*_*est*_ ≥ 0.

The direct method gives inconsistent *C* for the different *τ*_*est*_ > 0, as illustrated in Fig 8D to be compared with LO in A. Fig 8E measures the similarity between these matrices using the Pearson correlation coefficients between the weights corresponding to the mask in Fig 7D. LO (red) provides very consistent *C* for the different *τ*_*est*_, the heuristic method performs worse (purple) and the direct method (cyan) very poorly. The Σ consistency, however, is good for all methods. These findings are in line with the conclusions were found for artificial networks in Fig 5.

Each curve in Fig 8F represents a model whose *C* has been estimated using LO with ${\hat{Q}}^{0}$ only (green curve); ${\hat{Q}}^{0}$ and ${\hat{Q}}^{\tau }$ with *τ*_*est*_ = 2, 4 and 6 ms (blue to cyan). For each model, the *Q*^*τ*^ error between the model and empirical matrices is displayed for *τ* = *τ*_*mod*_ on the x-axis. The errors for the two darker blue curves are smaller than the green one for *τ*_*mod*_ ≥ 2 s, although the fit for *τ*_*mod*_ = 0 is slighly worse. Our optimization procedure thus improves the *whole* spatio-temporal *Q* fit, from ${\hat{Q}}^{0}$ to ${\hat{Q}}^{6}$, as compared to LO based on ${\hat{Q}}^{0}$ only. Directed connectivity is synonymous with capturing the information in the time-shifted ${\hat{Q}}^{\tau }$ with *τ* > 0. In the following, we take as reference for EC the *C* corresponding to *τ*_*est*_ = 4 s and 32% density in Fig 7F (and darkest blue curve in Fig 8F).

**Fig 9:**
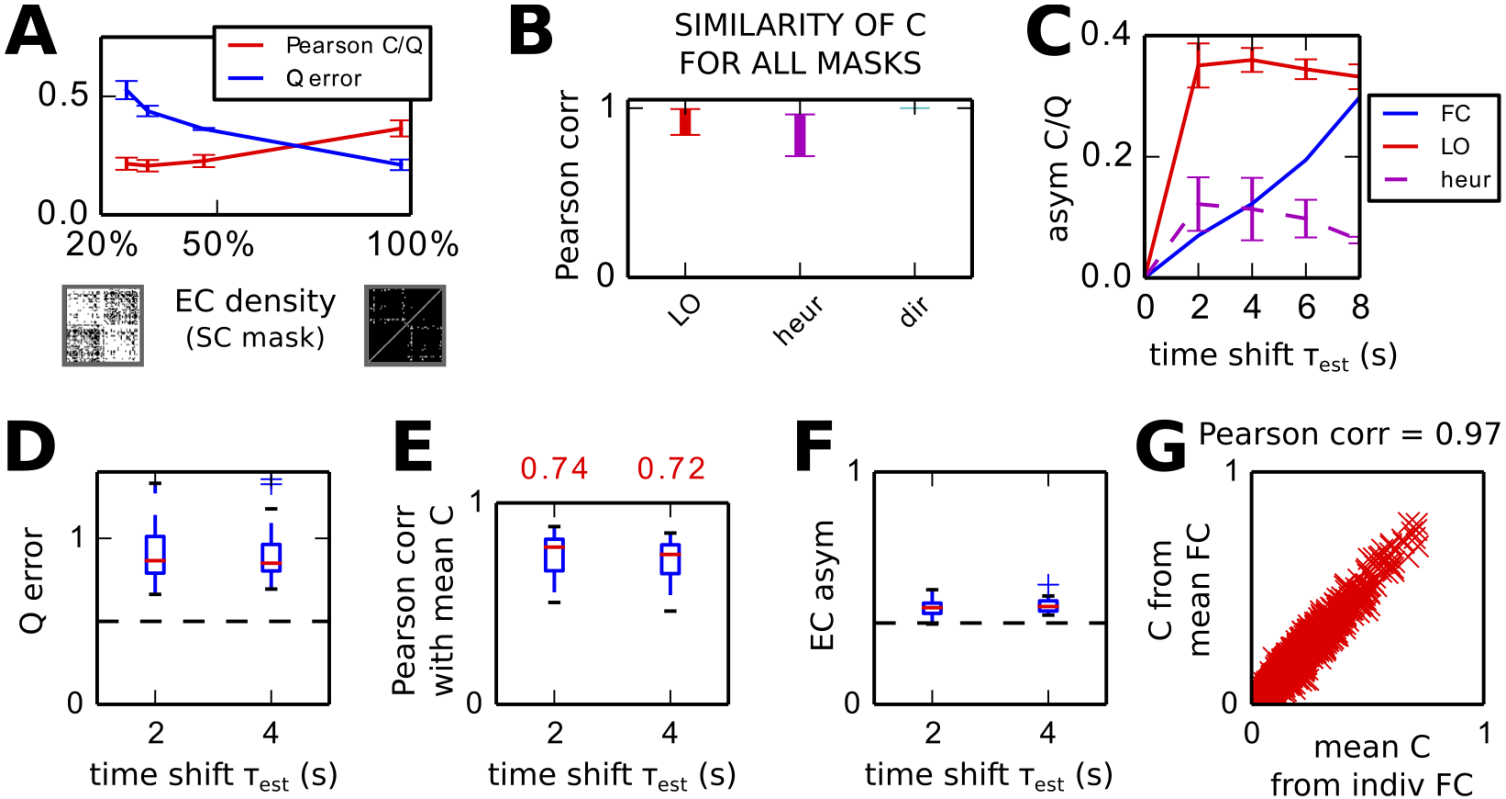
Robustness of estimated EC with respect to the EC density and individual FC. **A** Fit performance as a function of the density of EC. The blue curve indicates the *Q* error for optimizations with several masks for *C* (x-axis); the error bar correspond to the 4 *τ*_*est*_ > 0. The red curve indicates the similarity between the estimated *C* and the empirical ${\hat{Q}}^{0}$. **B** Similarity of estimated *C* measured by the Pearson correlation for the different masks in A and *τ*_*est*_ = 4 s. **C** Input-output asymmetry of the estimated *C* obtained for various optimization masks (error bars) and *τ*_*est*_ (x-axis). The red curve corresponds to LO and the purple curve to the heuristic method. The blue curve indicates the asymmetry of the empirical FC*τ*. **D** *Q* Error for LO based on individual FCs with *τ*_*est*_ = 2 and 4 s with 32% density. The horizontal dashed line corresponds to the *Q* error in Fig 7. **E** Similarity between the individual ECs and the EC obtained from the mean FC in Fig 7F. Similarity is measured by the Pearson correlation between the non-zero EC weights for the 32% density mask. **F** EC asymmetry of the individual ECs. The horizontal dashed line indicated the asymmetry of the EC in Fig 7F. **G** Agreement between the mean EC over individual FCs and the EC estimated from the mean FC.

To further check the consistency of the estimated *C*, we repeat the optimization procedures with various SC masks, similar to that in Fig 7D. By moving the threshold on the dwMRI values, we obtain various densities for the mask ranging from 25% to almost 100% in Fig 9A. Although the *Q* error decreases for denser *C*, the estimated *C* becomes more similar to $\hat{Q}$, and the mapping between *C* and *Q* more linear. Importantly, the estimated topology of *C* estimated by LO for the 4 densities is consistent, as measured by the Pearson correlation in red in Fig 9B. In comparison, the heuristic method is less consistent (purple). Likewise, Fig 9C shows that the asymmetry of the *C* matrices is consistent across SC masks and *τ*_*est*_ for LO (red), but less for the heuristic method (purple).

Finally, we perform LO for each of the 25 individual empirical FC. The *Q* errors are plotted in Fig 9D for *τ*_*est*_ = 2 and 4 s. The errors are similar for the two cases and are larger than the *Q* error for the average FC (dashed horizontal line). Fig 9E shows the good agreement between the individual ECs and the EC obtained from the mean FC in Fig 7F, with Pearson correlation coefficients around 0.7 for both *τ*_*est*_ = 2 and 4 s. As shown in Fig 9F, the asymmetry of the individual ECs is homogeneous around 0.4, which is slightly higher than the asymmetry of the EC from the mean FC that is equal to 0.35 (and indicated by the horizontal dashed line). Last, Fig 9G shows the excellent agreement between the mean of individual ECs and the EC from the mean FC. This confirms the consistency of the results obtained from our LO procedure.

### Interpretation of estimated cortical EC

**Fig 10:**
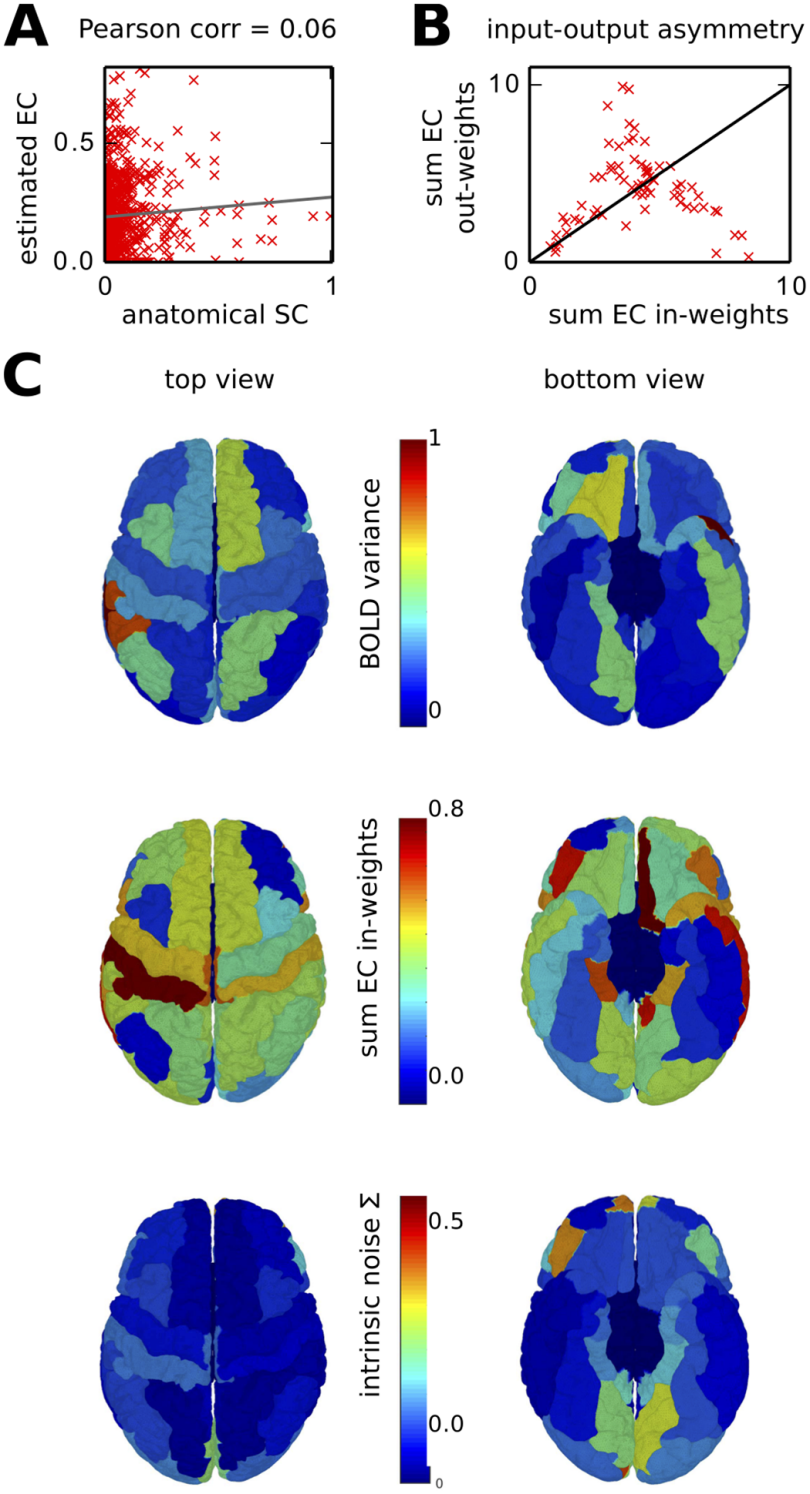
Interpretation of estimated cortical interactions and intrinsic noise. **A** Weak match between EC and SC values. Each red cross corresponds to a connection. **B** Plot of the sum of incoming *C* weights versus the sum of outgoing *C* weights (x-axis and y-axis, resp.) for each cortical area (red cross). **C** 3D mapped representations on the cortex of the variances of the empirical BOLD signal, sums of incoming weights in EC, and intrinsic noise (related to spontaneous noisy activity). Red corresponds to larger values and blue to small values.

Fig 10A shows the very weak match between the *C* and the SC corresponding to the dwMRI data averaged over all subjects, with a Pearson correlation coefficient equal to 0.06. The estimated EC is thus structurally very different from a scaled version of SC. As dwMRI reflects the density of cortico-cortical white-matter fibers, this suggests that the efficacies of these connections are determined by other factors than their size, such as types of neurotransmitters, concentration of synaptic receptors and excitability of cortical areas.

The estimated EC matrix in Fig 7F is not symmetric, meaning that cortical interactions are not reciprocal. This information is important as dwMRI data estimate the density of axonal fibers in the white matter, but do not recover the direction of those fibers. SC is by construction quasi symmetric, as is the case in Fig 7D. The matrix asymmetry for *C* relates to the reciprocity of intracortical connections and can be seen in the difference between incoming and outgoing strengths in Fig 10B. No area has both large incoming and outgoing weights, meaning that hubs act either as receptors or feeders.

In Fig 10C, the results are mapped onto the cortical surface. Cortical feeders with the largest outgoing weights are the left and right fusiform, middle temporal and superior temporal gyri, as well as the pre- and postcentral gyri in the left hemisphere. Cortical receivers with the largest incoming weights are the left and right precuneus, lateral occipital and superior parietal gyri, as well as the left isthmus of the cingulate gyrus. We also find that the following areas exhibit the largest values for Σ, synonymous with strong intrinsic variability: both left and right lingual gyri, pericalcarine cortices and frontal poles, as well as right cuneus and transverse temporal gyrus, and left pars orbitalis. This suggests the propagation of spontaneous activity, mainly from visual cortices and the prefrontal area.

## Discussion

We have shown in a noise-diffusion network model how the directed connectivity *C* can be retrieved from empirical covariances $\hat{Q}$. The key is to take into account the *temporal* information in covariances ${\hat{Q}}^{\tau }$ for non-zero time shifts *τ* > 0. Our proposed method gives a better fit of all ${\hat{Q}}^{\tau }$ for *τ* ≥ 0, not only ${\hat{Q}}^{0}$ that is often considered alone as an objective or goal in fitting procedures. Our theoretical study demonstrates two crucial requirements in order to recover the original *C* in the considered noise-diffusion networks: the time shift *τ*_*est*_ corresponding to ${\hat{Q}}^{\tau }$ must be matched with the time constant of the dynamical system *τ*_*x*_ estimated from the data (Fig 3); it is also necessary to adjust the diagonal matrix Σ that relates to spontaneous fluctuations experienced by each network node (Fig 4).

Our method provides an estimation of the asymmetry of intracortical connections (EC) from fMRI data combined with anatomical information from dwMRI. This is to our knowledge the first of its kind for the human connectome at the scale of the whole cortex. In addition to intracortical EC, our method also estimates via Σ the intrinsic variability of each cortical area, which is then shaped by EC to generate FC. This estimation relies on information in the BOLD spatio-temporal covariances, which convey information about the underlying neural connectivity (Fig 6). Our results suggest that the EC cortical hubs are either receivers or feeders, but not both (Fig 10).

### Inference of directed connectivity from observed network activity

It is known that zero-time-shift covariances are not sufficient to retrieve directed connectivity, but only its symmetric part [16, 25, 28]. Information-based methods able to estimate directionality such as likelihood maximization [13], Granger causality [14, 23] and transfer entropy [24] also use temporal information of the observed activity. In minimizing the matrix distance between the model *Q*^0/*τ*^ and objective ${\hat{Q}}^{0/\tau }$, instead of considering connections independently, the LO procedure captures network effects due to the recurrent feedback. Here we do not perform a stochastic gradient descent using many samples of the observed activity, but a deterministic optimization based on the ${\hat{Q}}^{0/\tau }$ averaged over the whole observation period (or several simulation sessions). It follows that the optimization is quick: a few minutes for 10^4^ optimization steps with a network of 50 nodes and a given *τ*_*est*_ on a recent desktop computer.

To obtain the best performance, we have shown that the time shift *τ*_*est*_ corresponding to ${\hat{Q}}^{\tau }$ used in LO and the time constant of the dynamical system *τ*_*x*_ should be matched. As shown in Fig 3E, poor estimation for large *τ*_*est*_ arises from the inaccuracy in empirical ${\hat{Q}}^{\tau }$; for small *τ*_*est*_, LO itself is unstable (see dashed curve in Fig 3F). Fig 5C shows that the method performance is not strongly affected by the network topology or the connectivity asymmetry, but worsens with the network size and becomes better with more observations used to calculate the empirical objective ${\hat{Q}}^{0/\tau }$. This is in line with previous results [25]. In addition to *C*, the intrinsic noise Σ received by the network nodes must be tuned to obtain a correct *C* estimation (Fig 4). Here we use a heuristic optimization for a diagonal Σ in Eq (20); the present framework should be extended to take into account correlated noise instead of white noise.

The direct method in Eq (8) to estimate *C* has been used previously with statistical tests to estimate the existence of connections from observed activity [16, 26]. As shown in Fig 5D, it does not work well for the level of noise in empirical observations ${\hat{Q}}^{0/\tau }$ considered here. This motivated the development of our LO procedure in Methods. Here we focus on the estimation of connection weights, i.e., their ranking; the detection of connections using statistical methods can be based on the estimated *C* from LO, but this is left for further work. Nevertheless, detection should be based on an as-good-as-possible estimated ranking of connections weights, which can be measured by the Pearson correlation. Our method for Ornstein-Uhlenbeck processes also bears similarities with MVAR [3], but we enforce additional constraints on the connectivity.

Recent studies [12, 28] have also used greedy algorithms to optimize symmetric *C* relying on ${\hat{Q}}^{0}$ using more elaborate network models. Those procedures update *C* step-by-step according to various measures such as the Pearson correlation between all matrix elements of zero-time-shift correlations. Here we have transposed this heuristic method to reproduce ${\hat{Q}}^{\tau }$ in addition to ${\hat{Q}}^{0}$, see Eq (21). Although the resulting *Q*^0/*τ*^ fit is close to perfect, the *C* estimation remains poor in Fig 5D. Taking the network effect correctly into account via LO is important to recover the original *C*, as compared to tuning connections individually based on the corresponding *Q*^0/*τ*^ value.

More generally, the problem with inferring *C* lies in the definition of observables or objective functions to constrain models without ambiguity: to a set of network parameters should correspond only a single value of the observable (here a pair ${\hat{Q}}^{0}$ + ${\hat{Q}}^{\tau }$). In [25], the minimization of matrix L1 norm for sparse networks was used to reduce the indetermination in using ${\hat{Q}}^{0}$ only. Beyond our results based on noise-diffusion processes, we expect that directed *C* can be recovered for other network models such as Hawkes processes (a.k.a. Poisson neurons) or binary neurons using second-order moments with non-zero time shift (here covariances). This is supported by recent results that demonstrate how the covariance structures are formally related across these neural models [38].

The present framework appears well suited to model activity as continuous signals; for spike trains generated by networks of GLM or Poisson neurons with simple temporal filters, it remains to be seen whether our fast tuning procedure can be adapted. We have only considered resting-state activity, but the procedure may also be extended to the case of multiple stimulation-response pairings. In particular, the external input *e* may be adjusted [16], in addition to *C* and Σ.

### Whole-cortex dynamic model fitted to fMRI data and cortical interactions

The goal of our model-based approach is to reproduce the resting-state FC obtained from fMRI. Although the ND model is not new, we propose a novel ‘LO’ procedure to tune the model parameters with suitable observables, namely time-shifted covariance matrices as FC. The estimated connectivity of the ND model relates to the intracortical EC, whose properties can then be analyzed [1]. For instance, EC can be searched for hubs, communities and similar features [30]. Here we have focused on hubs (Fig 10) and our results suggest that activity propagates from the visual, auditory and prefrontal areas. We find among the listening hubs the precuneus and superior parietal gyrus that belong to the default-mode network. These networks are usually found in resting-state FC and our results shed a new light on the architecture that shapes the activity propagation between them.

The ND model was previously used together with hemodynamics in order to reproduce similar fMRI data [12, 27]. As was demonstrated for that study for zero time shifts, the BOLD covariances with time shifts of the order of seconds also convey information about the interactions that determine the neural dynamics (Fig 6). This is in line with recent results showing that the BOLD time series convey information about cognitive processing for similar time lags [39, 40]. In our model, the EC asymmetry generates lags in covariances. Moreover, the LO procedure can estimate the EC ranking this neural connectivity from the BOLD covariances provided the ranking is preserved between the neural and the BOLD covariances for those more elaborate models involving a HRF (Fig 6). This supports the application of the ND model without HRF to model directly the BOLD data. In particular, the ND model seems well adapted to fMRI data at the considered parcellation of about 100 nodes: the BOLD autocovariances are close to exponential decays (i.e., straight lines for the log y-axis), as shown by the comparison between the ND model in Fig 1E and our experimental data for time shifts up to 8 s in Fig 7B. The corresponding time constants are rather homogeneous over all regions in Fig 7C, so we use a single *τ*_*x*_ = 5.3 s to calibrate the ND model, which corresponds to typical values for HRF [41]. In contrast, the autocovariances for the DMF+HRF in Fig 6C show a drop for *τ* ≥ 4 s that is not observed in the experimental data. We keep in mind that the precise relationship between the fluctuations of the BOLD signal and neural activity is still under debate [41–43]. Numerous studies such as those about repetition priming and suppression [44] have shown how changes in fMRI signals reflects those in neural activity, such as synchronization of the latter at a much shorter timescale. Moreover, fMRI is one of the few non-invasive methods to evaluate processing in the human brain (of course in vivo) and has many clinical applications [45], irrespective of its precise link to neural activity. This further supports efforts to develop generative models of BOLD and methods to better interpret these fMRI data.

An underlying assumption of our approach is that the individual variances of BOLD signals are meaningful [46]. This motivates the use of covariances to fit our model, rather than correlations that are often used [1]. The results in Fig 7 give a FC Pearson correlation coefficient (sometimes called predictive power) larger than 0.6 for *both* *Q*^0^ and *Q*^4^. In this sense, our study improves previous results [12, 31]. Importantly, the EC and Σ estimated by LO is surprisingly stable with respect to the choice of time shift for the considered experimental data. We find consistent results for a broad range of *τ*_*est*_ from 2 to 8 s, with a Pearson correlation coefficient larger than 0.9 in Fig 8. Choosing denser connectivity for EC improves the FC fit, but does not significantly change the EC ranking as shown in Fig 9A and 9B for results from 25% to 45% density. As an additional check, we have also shown that EC estimated from individual FCs coincides with EC obtained from the mean FC over all 25 subjects. In comparison, the heuristic and direct methods in Figs 8E and 9B and 9C give far more inconsistent results. Moreover, the heuristic method only uses part of the FC matrices when EC is sparse: it tends to overfit the existing connections compared to absent connections. A recent study [15] compared methods to estimate directed cortical interactions in a generative model of BOLD activity including hemodynamics: among those, Patel’s *τ*[47] gave the best performance, but that study was limited to sparse connectivity. Here we consider the FC for the whole cortex, which gives coupled constraints for a reasonably large network. Incorporating more areas limits the number of unknown contributions to the node activities, strengthening the estimation accuracy. This was observed for partial correlations in a similar manner for undirected connections [15].

Several previous studies used a scaled version of the SC matrix as EC [12, 28, 31]. As shown in Fig 8F, a symmetric EC does not satisfactorily fit time-shifted FCs. Furthermore, Fig 10A shows the weak match between dwMRI and our estimated EC values. This suggests that the fiber densities as measured by dwMRI may not be a good predictor for the dynamic interactions between cortical areas, but only for the skeleton of the cortical connectome. In addition, those previous studies used interhemispherical connections to improve the FC fit of the models. Here these connections also appear to be fairly strong in the estimated EC in Fig 7F, as shown by the two secondary diagonals in the top-left and bottom-right quadrant of the matrix. In any case, large covariances are obtained even for unconnected areas because of the strong overall feedback in the network, in line with results supporting that the cortex is in a state close to criticality [27]. As the dynamics of each cortical region has no nonlinearity in our model, large EC values are the only origin for the strong network effect, which induces the non-trivial mapping between EC and FC in Fig 7H.

As shown in Fig 1F for artificial networks, the *C* asymmetry is reflected in *Q*^*τ*^ for *τ* > 0. In our experimental data, the corresponding FC*τ* (blue line in Fig 9C) is not symmetric and relates to propagating activity [39]. In the ND network, the noise received by each area is embodied by Σ, which is then shaped by EC to generate FC. Here the LO procedure extracts the spatio-temporal FC information to estimate the EC asymmetry. The information about asymmetry in intracortical connections is thus important and complements the SC obtained from dwMRI, which is symmetric. Compared to previous studies that investigate the directionality of cortico-cortical connections [2, 4, 7], the novelty is that we estimate this property at the scale of the whole cortex. As all nodes have the same activation function, EC values indicate the relative interaction strengths between areas. What matters in our EC analysis is the ranking of EC weights: the difference between a low EC value and an absent connection is not so important here. Here we have estimated the asymmetry of cortico-cortical EC to be equal to 0.35 in Fig 8C. This is larger than macaque’s COCOMAC asymmetry that gives 0.14 for the same index [48, 49], but note that nonlinearity in the model dynamics would affect the precise EC values; what is important here is that EC is significantly asymmetric. This translates to an imbalance between incoming and outgoing EC weights. Fig 10B suggests that hubs are either feeders or receivers, but do not have both strong incoming or outgoing connections. As a conclusion, our work sets a suitable ground to study both local and global properties of the whole cortical connectivity, which will give insight in the underlying neural processing.
